# Early removal of the infrapatellar fat pad/synovium complex beneficially alters the pathogenesis of moderate stage idiopathic knee osteoarthritis in male Dunkin Hartley guinea pigs

**DOI:** 10.1186/s13075-022-02971-y

**Published:** 2022-12-28

**Authors:** Maryam F. Afzali, Lauren B. Radakovich, Madeline M. Sykes, Margaret A. Campbell, Kayley M. Patton, Joseph L. Sanford, Nicole Vigon, Ryan Ek, Gerardo E. Narez, Angela J. Marolf, Katie J. Sikes, Tammy L. Haut Donahue, Kelly S. Santangelo

**Affiliations:** 1 Department of Microbiology, Immunology and Pathology, Colorado State University, 200 West Lake Street, Fort Collins, CO 80523 USA; 2 Department of Biomedical Engineering, S631 Life Sciences Laboratory, University of Massachusetts, Amherst, 240 Thatcher Road, Amherst, MA 01003 USA; 3 Department of Environmental and Radiological Health Sciences, Colorado State University, 123 Flint Cancer Center, Fort Collins, CO 80523 USA; 4 Department of Clinical Sciences, Colorado State University, 1678 Clinical Sciences, Fort Collins, CO 80523 USA; 5 Biomedical Engineering Department, The University of Memphis, 3806 Norriswood, Memphis, TN 38152 USA

**Keywords:** Osteoarthritis, Infrapatellar fat pad/synovium complex, Gait, Inflammation, Biomechanics, Trace elements, Hartley guinea pig

## Abstract

**Background:**

The infrapatellar fat pad (IFP) is the largest adipose deposit in the knee; however, its contributions to the homeostasis of this organ remain undefined. To determine the influence of the IFP and its associated synovium (IFP/synovium complex or IFP/SC) on joint health, this study evaluated the progression of osteoarthritis (OA) following excision of this unit in a rodent model of naturally-occurring disease.

**Methods:**

Male Dunkin-Hartley guinea pigs (*n*=18) received surgical removal of the IFP in one knee at 3 months of age; contralateral knees received sham surgery as matched internal controls. Mobility and gait assessments were performed prior to IFP/SC removal and monthly thereafter. Animals were harvested at 7 months of age. Ten set of these knees were processed for microcomputed tomography (microCT), histopathology, transcript expression analyses, and immunohistochemistry (IHC); 8 sets of knees were dedicated to microCT and biomechanical testing (material properties of knee joints tissues and anterior drawer laxity).

**Results:**

Fibrous connective tissue (FCT) developed in place of the native adipose depot. Gait demonstrated no significant differences between IFP/SC removal and contralateral hindlimbs. MicroCT OA scores were improved in knees containing the FCT. Quantitatively, IFP/SC-containing knees had more osteophyte development and increased trabecular volume bone mineral density (vBMD) in femora and tibiae. Histopathology confirmed maintenance of articular cartilage structure, proteoglycan content, and chondrocyte cellularity in FCT-containing knees. Transcript analyses revealed decreased expression of adipose-related molecules and select inflammatory mediators in FCTs compared to IFP/SCs. This was verified via IHC for two key inflammatory agents. The medial articular cartilage in knees with native IFP/SCs showed an increase in equilibrium modulus, which correlated with increased amounts of magnesium and phosphorus.

**Discussion/conclusion:**

Formation of the FCT resulted in reduced OA-associated changes in both bone and cartilage. This benefit may be associated with: a decrease in inflammatory mediators at transcript and protein levels; and/or improved biomechanical properties. Thus, the IFP/SC may play a role in the pathogenesis of knee OA in this strain, with removal prior to disease onset appearing to have short-term benefits.

**Supplementary Information:**

The online version contains supplementary material available at 10.1186/s13075-022-02971-y.

## Introduction/background

Primary osteoarthritis (OA), particularly knee OA, currently burdens greater than 273 million people globally [1]. As a leading cause of pain and disability, OA is a major contributor to decreased quality of life [2]. Consequently, more than 1 million people undergo knee arthroscopy or joint replacement surgery each year due to end-stage OA in the United States [3], with the annual economic loss to Americans approaching $200 billion [4]. Unfortunately, there are no therapeutic regimens able to restore damaged cartilage to its normal phenotype or slow the progression of joint degeneration [5]. This reflects a need to improve our current understanding of the pathophysiology of the disease, which is associated with both inflammatory and biomechanical causes [6].

The knee is composed of many tissue types and structures, including several continuous but distinct adipose depots; specifically, the infrapatellar fat pad (IFP), the posterior knee fat pad, the quadriceps fat pad, and the pre-femoral fat pad [7]. The IFP is the largest of these and is found in the anterior aspect of the joint in the space shaped by the patella, femoral condyles, and tibial plateau. In spite of its bulk, the exact functions of the IFP and its associated synovium (IFP/synovium complex or IFP/SC) are not completely understood. The main role of the IFP is thought to be facilitating distribution of synovial fluid across the knee joint, thereby providing lubrication [8–10]. It also likely supplies shock absorbance from mechanical forces (similar to the menisci), knee joint stability, and may prevent instability and/or injury associated with loading forces to the knee joint [10, 11]. However, ex vivo work performed on cadavers revealed that resection of the IFP decreased tibial rotation of the knee [12]. From this, the authors extrapolated that the IFP may contribute to dictating the range of motion of the knee joint.

Despite this uncertainty, the IFP/SC is considered a player in overall knee joint homeostasis and there is evidence to support its part in the pathogenesis of knee joint OA [8–16]. Of note, the IFP is comprised of a network of adipocytes, fibroblasts, leukocytes (primarily macrophages and lymphocytes), and collagen matrix [7–9, 17]. As such, it is positioned to be a source of inflammatory mediators and/or immune modulators that may contribute to OA pathogenesis and associated pain [7–21]. For example, a study utilizing human tissues demonstrated that IFP-derived adipocyte conditioned media induced a pro-inflammatory response in T lymphocytes that resulted in increased proliferation and cytokine production [16]; Klein-Wieringa *et al.* [22] confirmed this pro-inflammatory phenotype in T cells and reported on mixed pro- and anti-inflammatory subtypes for IFP-related macrophages. In addition, Distal *et al.* have shown that interleukin (IL)-6 secretion from the IFP of women with knee OA was more than twice that of subcutaneous thigh fat [17]. Notably, Favero *et al.* revealed a similar increase in IL-6 in IFPs from OA patients, as well as vascular endothelial growth factor (VEGF) and monocyte chemoattractant protein-1 (MCP-1). Several excellent review articles are available that summarize the potential mechanisms linking the IFP/SC with OA [21, 23, 24].

The IFP/SC is not only affected by inflammation and pain with OA, but also shows evidence of hypertrophy, fibrosis, vascularization, and a decrease in size [16, 25, 26]; this can both alter, as well as respond to, the biomechanical properties of the collective joint [27]. Efforts to elucidate these specific changes include: finite element modeling of the knee, which identified the potential loss of typical stress-strain behavior of the IFP/SC in cases of OA [27]; computation biomechanics to explore the correlation between fat tissue structural conformation and mechanical behavior [28]; and custom indentation testing, which elucidated that the IFP from OA patients was a stiffer tissue depot relative to subcutaneous and visceral adipose, as well as heel fat pads [29].

Hoffa’s disease, sometimes called hoffitis, is a disease of the IFP (which has also been referred to as Hoffa’s fat pad). The pathophysiology of this disorder is not well documented; however, several mechanisms have been implicated, including acute trauma, microtrauma, and over-solicitation (repeated rotation and hyperextension) [30]. Regardless of the inciting cause, patients present with limited knee extension; magnetic resonance imaging highlights signal abnormalities in the IFP along the path of the adipose ligament [31–33]. Conservative approaches are proposed as a first-line of therapy. However, in scenarios where responses to these options are unsuccessful and/or the disease becomes chronic, arthroscopic resection/removal of the IFP/SC remains the next treatment of choice. In particular, individuals with this condition experience pain relief from undergoing arthroscopic subtotal removal of this adipose depot [34].

Given the above, we aimed to determine if unilateral removal of the IFP/SC would alter the pathogenesis and/or biomechanics of knee joint OA in an animal model of primary disease. The aims of this study were threefold: (1) to determine whether gait changes, an indication of potential symptom modification, would occur; (2) to assess potential disease modification; and (3) should disease modification be present, to evaluate both inflammatory and biomechanical outcomes that may be pertinent to this difference. We employed the Hartley guinea pig for this study, as it is a valuable rodent model of idiopathic OA. The majority of laboratory/rodent models of OA are artificially produced via surgery, chemicals, and/or genetic manipulation, and may not adequately recapitulate the predominant type of OA (non-traumatic or primary) found in people [35, 36]. The Hartley guinea pig, in contrast, is a natural disease model found in an outbred animal with OA histopathologic lesions similar to those seen in people [35, 36]. They are also identical to humans in their nutritional requirement for Vitamin C [35], which is key when considering pathology related to inflammation and oxidant activity. Pathology begins in the knee at 3 months of age, with mild pathology at 5 months, moderate OA at 7–9 months, and advanced OA documented at 12 months of age. We postulated that the IFP/SC acts as a local source of inflammation in this model [23] and that, by surgically removing it, we might improve OA outcomes by eliminating a source of negative mediators. Further, we were curious to determine whether the biomechanical testing or property outcomes would be influenced by this surgical intervention.

## Methods

### Experimental overview

All procedures were approved by the Institutional Animal Care and Use Committee and performed in accordance with the NIH Guide for the Care of Use of Laboratory Animals (#15-5854A; October 30, 2021). Animals (*n*=18) were maintained at Colorado State University’s Laboratory Animal Resources housing facilities and were monitored daily by a veterinarian. Guinea pigs were singly housed in solid bottom cages under a 12/12-h light dark cycle, at a temperature of 20°C–26°C and 30–70% humidity. Standard laboratory chow, hay cubes, and water were provided ad libitum. Sixteen Hartley guinea pigs of the same age, from a coincident but unrelated project, were utilized as an untreated control group for body weight comparisons. This study focused on male animals. A complementary study was performed in female guinea pigs and will be the focus of an independent manuscript.

Two sequential cohorts were utilized, as separate assessments were required for molecular-based properties of knee joints (*n*=10) versus biomechanical outcomes (*n*=8). For the former goal, the following evaluations were performed: treadmill-based gait collection; histopathology; clinical microcomputed tomography (microCT) grading; and transcript and protein expression. The sample size for this cohort was determined from a pilot study focused on the histologic assessment of OA as the primary outcome. Using a within-group error of 0.5 and an effect size of 1.0 in a Wilcoxon signed-rank test (matched pairs) for *t* tests on G*Power, power associated with an alpha level of 0.05 was calculated as 0.90 with a sample size of 10.

Biomechanical methods included: whole knee anterior drawer testing; patellar tendon pull to failure; indentation of articular cartilage and menisci; treadmill-based and voluntary gait assessment; quantitative microCT assessments; and atomic absorption spectroscopy (AAS) of articular cartilage. Sample size for this cohort was determined from a pilot study focused on indentation of articular cartilage as the primary outcome. Using the analysis provided above, but with an effect size of 1.2, power associated with an alpha level of 0.05 was calculated as 0.90 with a sample size of 8.

### Surgical removal of the IFP

Resection of the IFP/SC was performed on all animals (*n*=18). After medial parapatellar arthrotomy of the right knee, the patella was temporarily displaced cranially with the knee in extension to permit access to the femoral groove. The patella was repositioned once the IFP/SC was exposed medially for removal; the skin incision was closed following full dissection. While care was taken to dissect only the IFP from the right knee of these animals, it cannot be excluded that portions of the synovium were removed. An identical sham procedure, with minor manipulation but without removal of the IFP/SC, was performed on the left knee and served as a matched internal control for each animal.

### Gait assessments

Obligatory treadmill-based gait analysis was performed on all animals (*n*=18) using a DigiGait^TM^ treadmill system (Mouse Specifics, Inc., Framingham, MA). Additionally, animals dedicated to biomechanical testing (*n*=8) performed voluntary weight-bearing assessment using a Rodent Walkway System (Tekscan, South Boston, MA). Animals were acclimated to both apparatuses over a 2-week period. Data collection was performed by the same handlers during the same time period (8:00AM to 12:00PM); the order in which animals were analyzed was randomly selected. Baseline gait and weight-bearing analyses were performed the day before IFP removal surgery. Subsequent data were collected every 4 weeks after surgery, with the final time point occurring the day before termination. Data is absent for 8 guinea pigs during week 12 due to COVID-19 pandemic restrictions.

### Tissue collection

All animals (*n*=18) were harvested at 7 months of age. Body weights were recorded at the time of harvest; animals were transferred to a CO_2_ chamber for euthanasia. Hind limbs were removed at the coxofemoral joint. The lengths of the left and right femurs were measured using digital calipers. Animals in the first cohort (*n*=10) had their left and right hind limbs removed and placed in 10% neutral buffered formalin for 48 h and transferred to phosphate buffer saline (PBS) for microCT grading. After microCT, limbs were placed in a 12.5% solution of ethylenediaminetetraacetic acid (EDTA) at pH 7 for decalcification. EDTA was replaced twice weekly for 6 weeks. Animals utilized for biomechanical outcomes (*n*=8) had their left and right hind limbs immediately frozen and stored at −20°C for quantitative microCT followed immediately by biomechanical assessments.

### MicroCT

Knee joints from the first cohort (*n*=10) were scanned in PBS using the Inveon microPET/CT system (Siemens Medical Solutions, Malvern PA), with a voxel size of 18 μm, a voltage of 100 kV, and an exposure time of 1356 ms. Clinical features of boney changes of OA were scored using a published whole joint grading scheme [37]. Features graded included the presence/size and location of osteophytes, subchondral bone cystic changes, subchondral bone sclerosis, articular bone lysis, and intraarticular soft tissue mineralization. Images were scored by a board-certified veterinary radiologist (AJM) blinded to limb identification.

Knees dedicated to biomechanical outcomes (*n*=8) were scanned in PBS for microCT using a Bruker Skyscan 1276 (Bruker, Billerica, MA) with a voxel size of 20 μm, a voltage of 100kV, and an exposure time of 473 ms. Osteophytes were identified by manually outlining new bone growth every 10 slides by a single operator (GEN). Osteophytic bone volume was identified by reduced bone mineral density and as being outside the original joint margins using CTAn software. Similar to previous studies, four regions of interest (ROI) were chosen to coincide with areas of mechanical testing on the articular cartilage of the lateral and medial compartments [38–41]. Trabecular volumes of interests (VOIs) ranged from just below the cortical bone to the growth plate. CTAn software (Bruker, Billerica, MA) was used to determine bone volume/ tissue volume (BV/TV), trabecular thickness (Tb.Th), trabecular separation (Tb.Sp), trabecular number (Tb.N), and volumetric bone mineral density (vBMD). Cortical bone VOIs were located between the joint margin and trabecular bone and were identified by a single operator (NV) using CTAn software. Cortical bone mineral density, cortical bone porosity, and cortical thickness were determined using CTAn.

### Histologic grading of OA

Following decalcification and paraffin-embedding of knees from the first cohort (*n*=10), three sagittal 5-μm sections were made through each knee joint: (i) mid-sagittal slices were used for histologic evaluation of the IFP/SC; and sagittal slices through both the (ii) medial and (iii) lateral compartments were utilized to assess OA changes in four sites (medial tibia, lateral tibia, medial femur, and lateral femur). The mid-sagittal sections were stained with hematoxylin and eosin (H&E) and Masson’s trichrome stain to confirm structural modifications. The medial and lateral compartments were stained with toluidine blue for OA grading using OARSI recommended criteria [36]. The OARSI published grading scheme is based upon species-specific features of OA, including articular cartilage structure, proteoglycan content, cellularity, and tidemarks integrity. Two blinded veterinary pathologists (LBR and KSS) performed histological grading. Scores from each of the four anatomic locations were summed to obtain a total knee joint OA score for each right and left hind limb. An intraclass correlation coefficient of 0.9 for between-reviewer consistency was calculated; the few minor discrepancies identified were resolved prior to statistical analysis.

### Biomechanical analyses

A servo-hydraulic testing system (MTS Corp, Eden Praire, MN) and 9-N load cell (Futek, Irvine, CA) were utilized for indentation relaxation tests. Cartilage and menisci from both native IFP/SC and FCT knee joints were subjected to indentation relaxation tests in a phosphate-buffered saline bath to determine instantaneous and equilibrium moduli. The bath was mounted to a two-degree-of-freedom camera mount and an X-Y stage to allow for the indentation surface to be oriented normally to the indenter tip. Both the femoral and tibial cartilage were indented on the medial and lateral plateaus, which is a location where there would be cartilage on cartilage contact. To test the cartilage, the long bones were potted distally and adjusted to indent perpendicular to the surface. The cartilage was assumed to be 0.5 mm thick on the femur and 0.75 mm on the tibia. The menisci were extracted off the tibial plateau at the anterior and posterior root attachments. They were then glued onto a plexiglass mounting plate to allow the proximal surface to be mounted perpendicular to the indenter tip. The medial and lateral menisci were indented at both the anterior and posterior locations. Menisci were assumed to be 1.2 mm thick and, following a preload of 50mN, menisci were indented to 10% strain (0.12 mm) in 1 s and held for 900 s to reach equilibrium. Hertzian contact between an elastic half space (meniscus) and a rigid sphere (indenter) was assumed [42–44] A custom MATLAB algorithm (Mathworks, Natick, MA) was used to calculate the instantaneous and equilibrium moduli from the collected data [42–44]. The algorithm searched for the peak instantaneous load response and divided by cross-sectional area to obtain instantaneous modulus. It then determined when equilibrium was reached (when the load did not change by more than 1% over 5 minutes); that load was divided by cross-sectional area to determine equilibrium modulus.

### Atomic absorption spectroscopy (AAS)

Phosphorus (P), magnesium (Mg), calcium (Ca), zinc (Zn), and iron (Fe) quantification were performed on samples of tissues from the second cohort (*n*=8), including the native IFP/SC or replacement tissue that developed in its place; articular cartilage from the tibia and femur (representing pooled medial and lateral compartments); and separate distal medial and lateral femoral condyles, representing combined cortical and subchondral trabecular bone. Briefly, dried tissue was weighed, reduced to ash, sonicated in 3.6N nitric acid, and diluted 30-fold with deionized water [45]. Diluted samples were analyzed using a Model 240 AA flame atomic absorption spectrometer and SpectraAA software (Agilent Technologies, Santa Clara, CA) [45]. Mineral levels were reported as parts per million (ppm) dry weight (dw) [45].

### Gene expression using nanostring technology

Total ribonucleic acid (RNA) was isolated from either the IFP/SC or replacement tissue that remained in formalin-fixed paraffin-embedded blocks (*n*=9) following acquisition of adequate sections for histopathology and immunohistochemistry (IHC) using a commercially available kit specific for such (Roche, Basel, Switzerland). A custom set of guinea pig-specific probes were designed and manufactured by NanoString Technologies (Seattle, WA) for the following genes: adiponectin (ADIPOQ), complement component 3 (C3), catalase (CAT), collagen type 1 alpha 2 (COL1A2), fatty acid synthetase (FASN), G protein pathway suppressor 2 (GPS2), leptin (LEP), MCP-1, and matrix metallopeptidase-2 (MMP-2), nuclear factor kappa-β transcription complex (NF-kB p65 and NF-kB p50), and nuclear receptor subfamily 4 group A member 2 (NR4A2) (Supplemental Table 1). Per initial RNA quantification (Invitrogen Qubit 2.0 Fluorometer and RNA High Sensitivity Assay Kit, Thermo Fisher Scientific, Waltham, MA) and Fragment Analyzer quality control subsets (Fragment Analyzer Automated CE System and High Sensitivity RNA Assay Kit, Agilent Technologies), the optimal amount of total RNA (800.00ng) was hybridized with the custom code-set in an overnight incubation set to 65°C, followed by processing on the NanoString nCounter FLEX Analysis system. Results were reported as absolute transcript counts normalized to positive controls and three housekeeping genes (β-actin, succinate dehydrogenase, and glyceraldehyde-3-phosphate dehydrogenase). Any potential sample input variance was corrected by the use of the housekeeping genes and the application of a sample-specific correction factor to all target probes. Data analysis was conducted using the nSolver software (NanoString Technologies).

### IHC and quantitative analysis

IHC was performed on mid-sagittal sections containing medial tibial cartilage, the IFP/SC, or replacement tissue using polyclonal rabbit antibodies to MCP-1 (Abcam ab9669) or NF-kB p65 (Abcam ab86299), each at a concentration of 2.5 μg/ml (1:400 dilution). Prior to staining, slides were incubated in citrate buffer for 5 h at 55°C for antigen retrieval, as recommended for skeletal tissues [46]. Normal goat serum (2.5%) was used as a blocking reagent. Slides were incubated in primary antibody overnight in a humidified chamber at 4°C, followed by a 30-minute incubation with a biotinylated goat anti-rabbit secondary antibody (1:200). Bone marrow hematopoietic cells and macrophages served as internal positive controls for each section. Negative assay controls — rabbit immunoglobulin at 2.5 μg/ml and secondary antibody (1:100), alone — did not result in background immunostaining. Sections were counterstained with hematoxylin, cover slipped, and imaged by light microscopy. Data was quantified as integrated optical density using ImagePro-Plus 7 Software (Media Cybernetics, Rockville, MD). Four 1-mm-wide regions of interest of medial tibial cartilage and replacement tissue were analyzed for MCP-1 and NF-κkB p65 expression; data for each tissue type was averaged prior to statistical analysis.

### Statistical analyses

Rationale for excluding individual values from data sets was determined prior to analysis and included whether an appropriate sample was unable to be collected, did not pass quality control parameters, or if integrity was compromised. Exclusion of animals resulted in *n*=12 animals for treadmill-based gait results; *n*=8 for OARSI scoring; *n*=9 for transcript expression; *n*=8 for MCP-1 cartilage IHC; *n*=6 for NF-kB p65 cartilage IHC; *n*=9 for MCP-1 and NF-kB p65 IFP/SC or replacement tissue IHC; *n*=7 for biomechanical analyses; and *n*=5 for AAS of IFP/SC or replacement tissue. Complete data sets were available for all other outcomes.

Statistical analyses were performed with GraphPad Prism 9.4.1(La Jolla, CA) with significance set at P< 0.05. Data underwent normality and variance testing with the Shapiro-Wilk test. For mobility outcomes, longitudinal data was analyzed using two-way or mixed model ANOVA with a Tukey post hoc test analysis. For all other analyses, normally distributed data with similar variance were compared using paired t-test^†^ for normally distributed data; Wilcoxon matched-pairs signed rank test^×^ was used for non-normally distributed data.

## Results

### General description of study animals

All guinea pigs remained clinically healthy throughout the study (Supplemental Fig. 1A). There was no significant difference in body weight when this cohort was compared to 7-month-old Hartley guinea pigs utilized as untreated controls in a parallel but unrelated study (Supplemental Fig. 1B). Mean total body weight (terminal weights) was 1141 g (95% CI: 1083–1199 g) in the IFP removal group and 1100 g (95% CI: 1070–1130 g) in the control group. To ensure that potential differences in gait were not attributable to changes in skeletal properties, left and right femur lengths from all IFP/SC removal guinea pigs were measured. Femur lengths between the left [sham control (mean=43.92 mm; 95% CI: 42.85–44.99 mm)] and right [IFP/SC removal (mean= 43.94; 95% CI: 42.89–45.00 mm)] hind limbs were not significantly different (*P* = 0.2871) (Supplemental Fig. 1C).

### Gait analyses

**Fig. 1:**
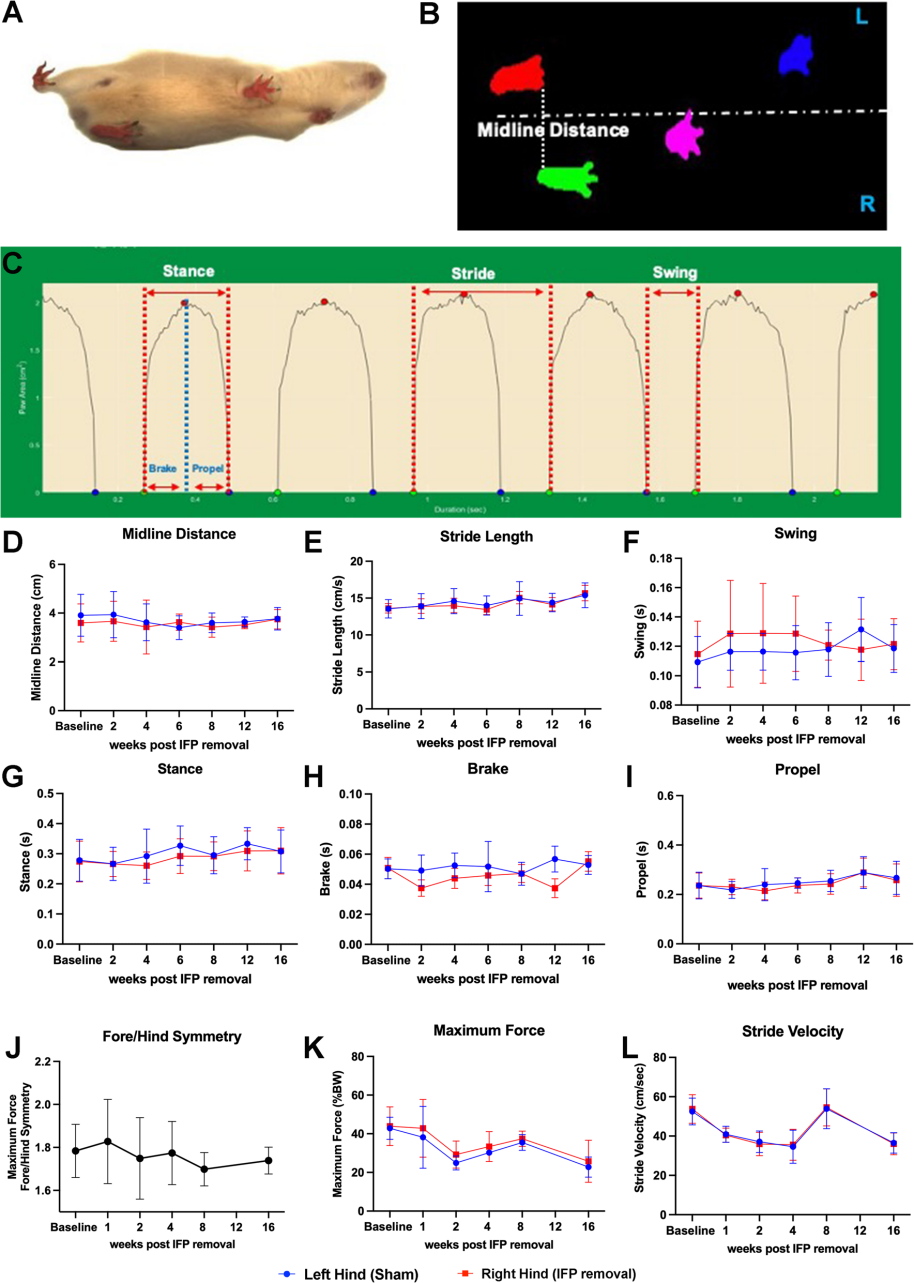
Mobility assessments. (**A**) Schematic of treadmill-based gait analysis measurements. (**B**) Video still image showing a guinea pig running on a transparent treadmill (viewed from below) at 45 cm/s. Placement for each paw is detected from the video illustrated in **A**. (**C**) Paw area in contact with treadmill surface over time for a representative single paw (left hind limb), from which multiple stride indices can be obtained (labeled). Longitudinal data of IFP (blue) and replacement tissue (red) contralateral limb measurements of midline distance (**D**), stride length (**E**), swing (**F**), stance (**G**), brake (**H**), and propel time (**I**). Voluntary weight-bearing assessments between fore/hind symmetry (**J**), maximum force (**K**), and stride velocity (**L**). *P*-values represent the significance of two-way ANOVA (or mixed model) with Tukey’s post hoc test analyses

To assess whether the removal of the IFP impacted the gait of each animal, we contrasted parameters for each matched hindlimb. The ventral view of a guinea pig (Fig. 1A), digital video images (Fig. 1B), and representative dynamic gait signals (Fig. 1C) of a guinea pig walking on a transparent treadmill belt at 45 cm/s are provided. No differences in midline distance (Fig. 1D), stride length (Fig. 1E), swing (Fig. 1F), stance (Fig. 1G), brake (Fig. 1H), or propel (Fig. 1I) were seen between IFP/SC removal and contralateral (native IFP) hindlimbs. Significant differences were noted over time in stride length, stance, and propel values (*P* < 0.0001). Voluntary weight-bearing assessment confirmed no difference between fore/hind symmetry (Fig. 1J), maximum force (Fig. 1K), or stride velocity (Fig. 1L) between hind limbs. Additional voluntary weight-bearing parameters are provided in Supplemental Table 2.

## Characterization of IFP versus replacement tissue

### Morphologic description of H&E and Masson trichrome stained slides

**Fig. 2:**
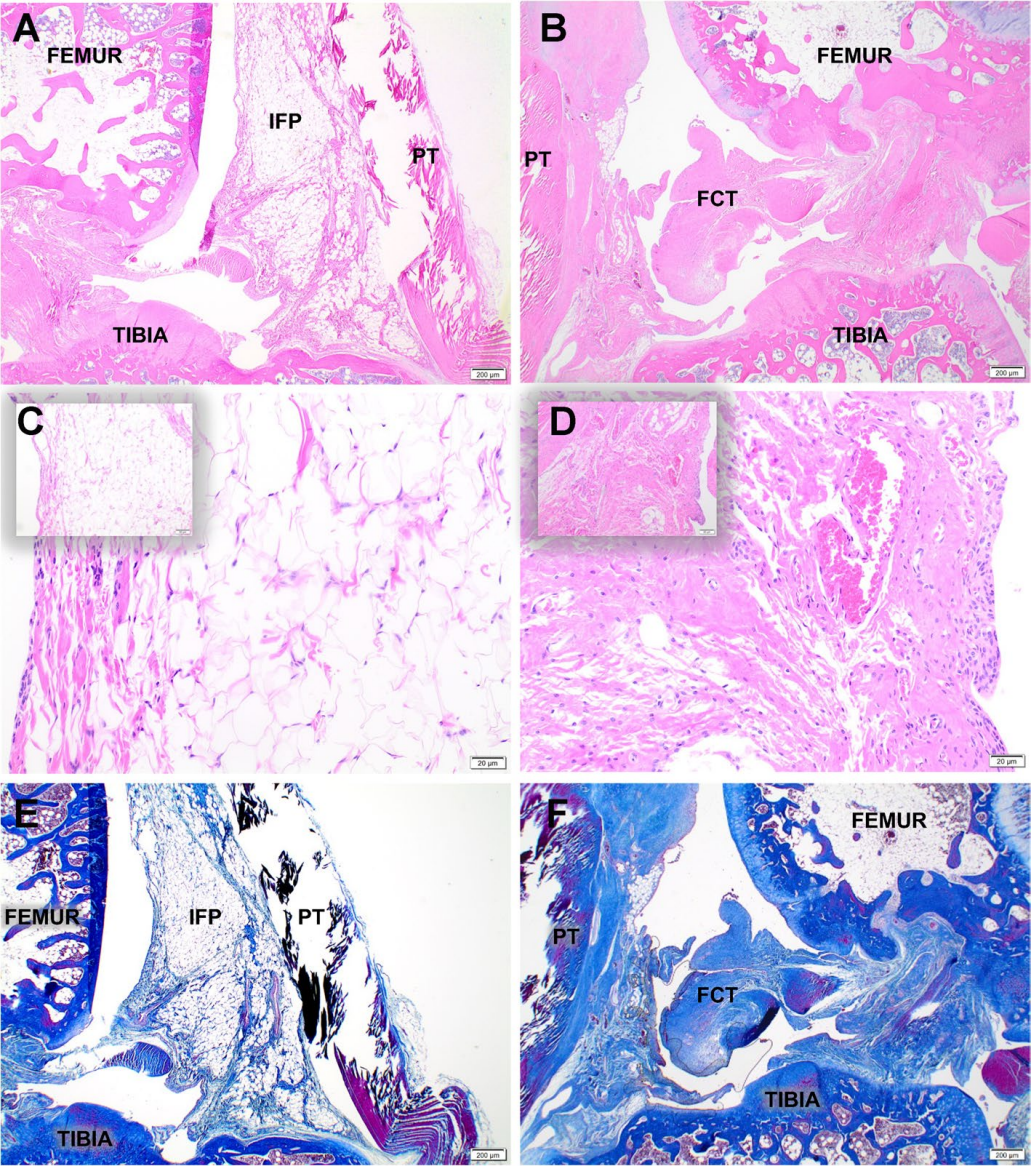
Characterization of IFP/SC and replacement tissue. Representative mid-sagittal photomicrographs of a stifle joint from a control (**A**, **E**) and IFP/SC removal (**B**, **F**) guinea pig; H&E (**A**, **B**), and Masson’s trichrome (**E**, **F**), 2× objective. (**A**, **C**) Control knee joint from a 7-month-old guinea pig depicting the normal histoanatomic location of the IFP/SC in its native state. (**B**, **D**) Knee joint from 7-month-old guinea pig four months after IFP/SC removal. The IFP/SC (**C**) is replaced with dense fibrous connective tissue (FCT) 20× objective for the main photo; 10× objective for inset (**D**)

Hematoxylin and eosin (H&E) staining confirmed that left (surgery sham control) knees retained the typical histological properties of IFP/SC, including mature adipocytes, a stromal vascular fraction, and white blood cells (predominantly large and small mononuclear cells consistent with macrophages and lymphocytes, respectively) (Fig. 2A, C). In contrast, the right (IFP/SC removal) hindlimbs exhibited a development of a thick band of dense fibrous connective tissue (FCT) in the space previously occupied by the native IFP/SC (Fig. 2B, D). Further histological examination with Masson trichrome stain confirmed the increased collagenous nature of the FCT compared to the native IFP (Fig. 2E, F; Supplemental Fig. 2). Notably, intact synovium as part of the IFP/SC was confirmed on histopathology for all limbs at termination.

### Biomechanical analyses

Cranial/anterior drawer and pull to failure assessment did not provide any significant changes when IFP/SC was compared to FCT (Supplemental Table 3). As the cranial/anterior drawer test is a measure of the cranial/anterior cruciate integrity, these findings were anticipated.

### Transcript and protein expression analyses

#### Components of adipose tissue

**Fig. 3:**
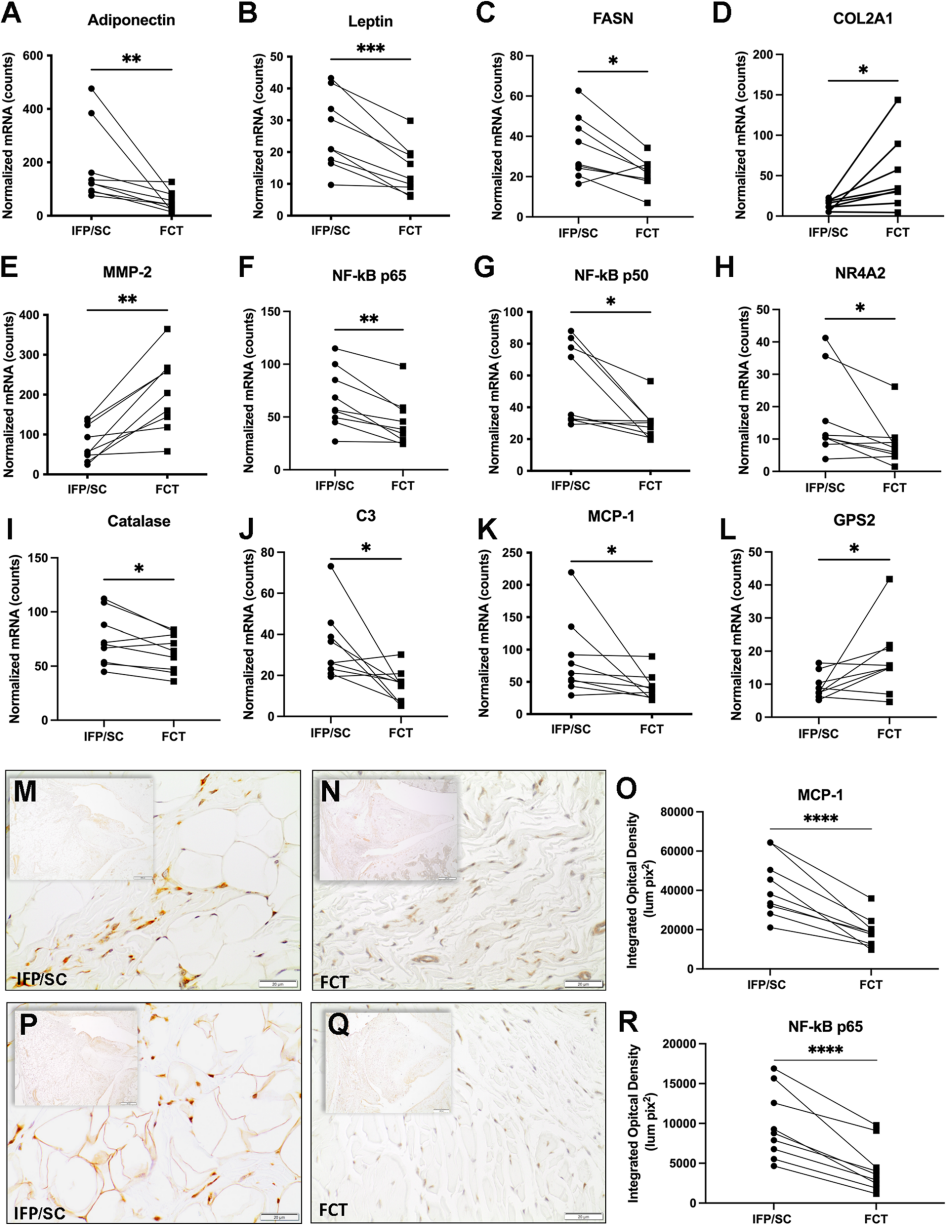
Transcript and protein expression analyses. Normalized mRNA counts for adiponectin (**A**), leptin (**B**), FASN (**C**), COL2A1 (**D**), MMP-2 (**E**), NF-kB p65 (**F**), NF-kBp50 (**G**), NR4A2 (**H**), catalase (**I**), C3 (**J**), MCP-1 (**K**), and GPS2 (**L**) in IFP/SC and FCT of knee joints. Immunohistochemistry for MCP-1 expression in IFP/SC  (**M**; left hind limb) and FCT (**N**; right hind limb). MCP-1 is a cytokine regulated by the NF-kBp65 pathway; expression in the FCT was reduced compared to the native IFP/SC. (**O)** Figure representing quantitative analysis of MCP-1-stained tissue subtracted from IgG control tissue for all samples. NF-kB is a transcription factor that is known for regulating inflammatory responses within inflammatory cells; protein expression is increased in IFP/SC (**P**; left hind limb) versus FCT (**Q**; right hind limb). (**R**) Figure representing quantitative analysis of p65 stained tissue subtracted from IgG control tissue for all samples. P-values were determined by paired t-test^†^ for non-parametric Wilcoxon test^×^ for non-normally distributed data. ***P <0.0005, **P<0.005, and *P<0.05

As was expected given the histologic findings, mRNA levels revealed that the FCT had a lower expression of transcripts for ADIPOQ (*P* = 0.0039), LEP (*P* = 0.0005), and FASN (*P*= 0.0130) compared to the native IFP/SC (Fig. 3A–C).

#### Inflammatory/degradative mediators

Compared to the IFP/SC, the FCT had decreased expression of mRNA for NF-kB p65 (*P*= 0.0021), NF-kB p50 (*P*=0.0117), NR4A2 (*P*=0.0273), CAT (*P*=0.0273), C3 (*P*=0.0195), MCP-1 (*P*=0.0117). COL2A1 (*P*=0.0178), MMP-2 (*P*=0.0018), and GPS2 (*P*=0.0489) were increased (Fig. 3D–L). The complete custom gene panel is provided in Supplemental Table 4.

### IHC for local inflammatory responses

To confirm the transcript expression data, MCP-1 and NF-kB p65 at the protein level were evaluated to assess local inflammation in the native IFP/SC versus the FCT (Fig. 3M–R). Similar to the above, immunostaining of both proteins was significantly lower in the FCT (*P*<0.0001).

### Atomic absorption spectroscopy of trace element in IFP/SC vs FCT

Trace element concentrations were not significantly different between the IFP/SC and FCT (Supplemental Table 5).

## Characterization of bone and cartilage pathology

### MicroCT

#### Clinical whole joint OA scores

**Fig. 4:**
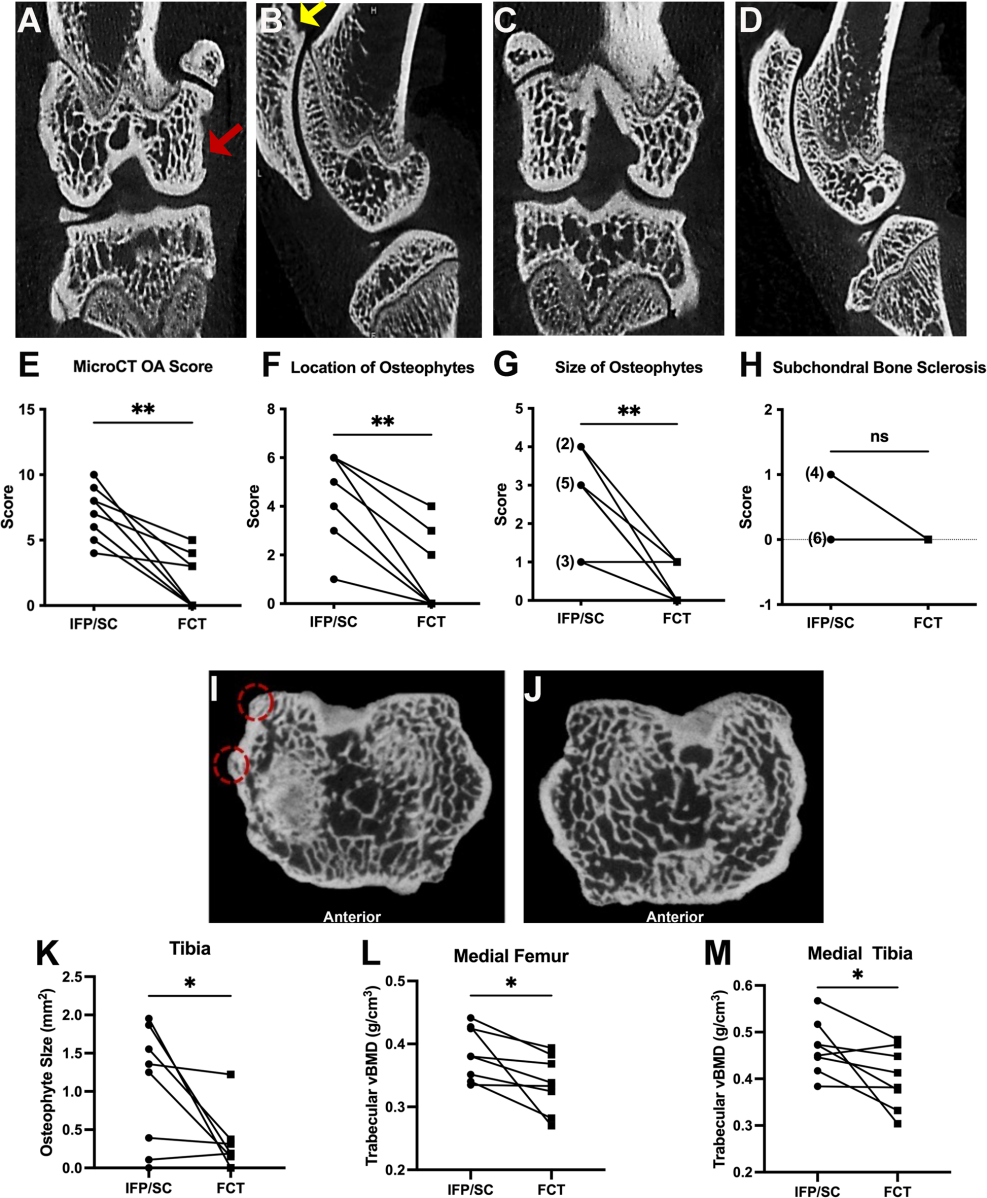
Clinical and quantitative MicroCT. Representative photos from microCT evaluation of knee joints using the published scoring system. (**A**) Coronal and sagittal (**B**) sections of an IFP/SC-containing knee. Subchondral bone sclerosis is present on the medial femoral condyle (coronal section, red arrow). An osteophyte is highlighted on the proximal patella (sagittal section, yellow arrow). (**C**) Coronal and sagittal sections of the FCT limb, which is relatively unaffected. (**E**) The clinical microCT OA score demonstrated a significant decrease in bony changes in the FCT knees (*P*= 0.0020). Contributions to whole joint microCT OA score components included location of osteophytes (**F**), size of osteophytes (**G**), and subchondral bone sclerosis (**H**). Cross-section of tibiae containing either IFP/SC (**I**) or FCT (**J**); osteophytes are highlighted on the medial posterior tibia (red dashed circles). (**K**) Osteophyte volume mm^3^ was significantly decreased in FCT versus IFP/SC knees. Trabecular vBMD (g/cm^3^) of the medial femora (**L**) and tibiae (**M**) were decreased by the FCT when compared to the IFP/SC. Normally distributed data with similar variance were compared using parametric *t* test^†^. Data with non-Gaussian distribution were compared using non-parametric Wilcoxon^×^ matched – pairs signed rank test. **P<0.005 and *P<0.05

Whole joint microCT OA scores provide a comprehensive assessment of bony changes observed in the tibia, femur, and patella of each animal. All IFP/SC hind limbs presented with a mixture of small (< 1mm) and/or large (> 1mm) osteophytes at multiple anatomical locations (medial and/or lateral tibia, patella, or femur) with 9 out of 10 of these knees having osteophytes in two or more locations (Fig. 4B, F, G). In addition, 4 of the 10 IFP/SC knees demonstrated subchondral bone sclerosis, as shown in Fig. 4A and H. For FCT knees, only 3 out of 10 animals had small osteophytes present; of these 3 animals, 2 animals had small osteophytes on either the patella or femur, with 1 animal having osteophytes on both the tibia and femur (Fig. 4D, F, G). Of note, no evidence of articular bone lysis or mineralized intra-articular soft tissue was present within any knee joints. Cumulatively, OA scores were significantly higher in knees that contained the native IFP/SC (range of 5 to 10) versus those with the replacement FCT (range of 0 to 5; *P* = 0.0020) (Fig. 4E).

#### Quantitative microCT and trace elements

The 8 guinea pigs dedicated to the biomechanical testing were analyzed for quantitative outcomes. Volumes of osteophytes (mm^3^) on reconstructed femurs, tibias, and patellas were measured. A representative microCT section presents osteophytes on the medial posterior tibia of the IFP/SC limb (Fig. 4I, denoted by red dashed circles); in contrast, the FCT limb of the same animal did not have this same osteophyte formation (Fig. 4J). Quantitative measurements confirmed a significant decrease of mean osteophyte volume 1.060 mm^3^ (95% CI: 0.000–1.223) vs FCT mean 0.3047 mm^3^ (95% CI: 0.000–1.223) (Fig. 4K; *P*=0.0469).

Mean values (and 95% confidence intervals) for each of the quantitative microCT measurements can be found in Supplemental Table 6. Significant differences between the native IFP/SC and FCT were found for: trabecular vBMD of the femur (Fig. 4L; *P*=0.0297) and tibia (Fig. 4M; *P*=0.0483).

To investigate a potential explanation for the increase in vBMD for IFP/SC-related subchondral trabecular bone, trace elements were measured in separate distal medial and lateral femoral condyles, representing both cortical and subchondral trabecular bone (Supplemental Table 7). Of interest, the following differences were present in medial femoral condyles: trabecular bone from IFP/SC-containing knees had significantly decreased P (*P*=0.0469), significantly increased Mg (*P*=0.0059), and a trending increase in Ca (*P*=0.0777).

### OARSI histology score

**Fig. 5:**
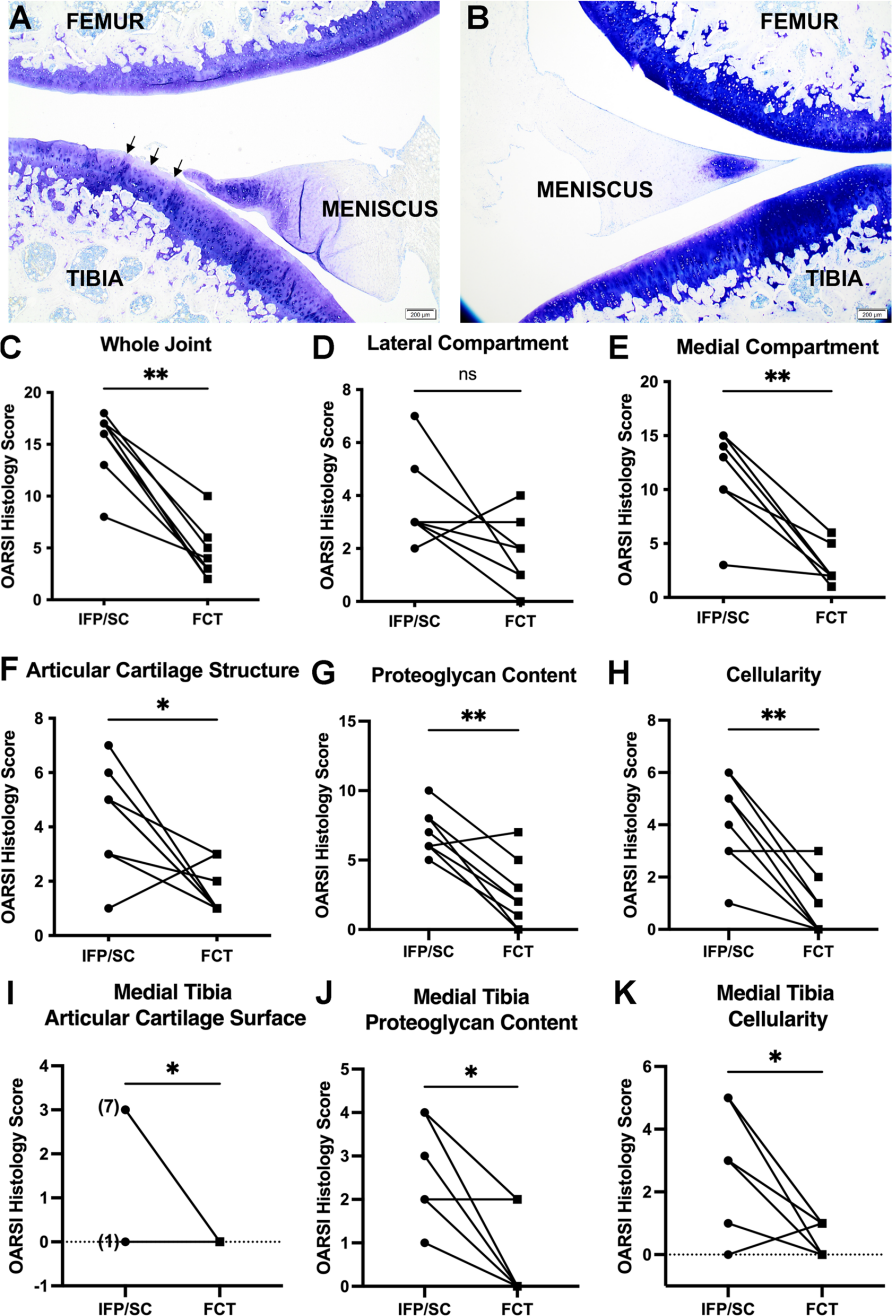
OARSI Score. Representative photomicrographs of toluidine blue-stained sections from medial compartments for OARSI scoring. (**A**) Irregular articular surface with mild fibrillation and proteoglycan loss is present in the superficial zone of the tibia from the knee containing the native IFP/SC. (**B**) The contralateral knee from the same animal exhibits a smooth cartilage surface and very mild proteoglycan loss. (**C**) OARSI whole joint OA score confirmed a significant statistical difference in cartilage and proteoglycan change. Contributions to whole joint OARSI score components included the lateral compartment (**D**) OARSI score, which was not significantly different, and medial compartment (**E**) mean OARSI histologic score, which was significantly different. Contributions to the medial compartment included articular cartilage structure (**F**), proteoglycan content (**G**), and cellularity (**H**). More specifically, medial tibia OARSI score of articular cartilage surface (**I**), proteoglycan content (**J**), and cellularity (**K**) contributed to the significant decrease in histolopathology by the FCT when compared to the IFP/SC. Normally distributed data with similar variance were compared using parametric *t* test^†^. Data with non-Gaussian distribution were compared using non-parametric Wilcoxon^×^ matched – pairs signed rank test. **P<0.005 and *P<0.05

Representative lesions of both knees from the same animal are shown in Fig. 5A and B. Histologic OA scores were significantly higher in IFP/SC group compared to FCT-containing knees (Fig. 5C; *P*=0.004). When medial and lateral compartments were analyzed independently, the lateral compartment did not have a significant difference (Fig. 5D) while the medial compartment did demonstrate a significant decrease in OARSI score in the FCT knee when compared to IFP/SC (Fig. 5E). Figure 5A shows and irregular, undulated cartilage surface, with mild fibrillation and proteoglycan loss in the superficial zone of the tibia from the knee with the native IFP/SC. Figure 5B demonstrates a smooth cartilage surface and very mild proteoglycan loss in the knee with the FCT. Overall, the lower histological OA scores for the hind limbs that underwent IFP/SC removal confirmed a maintenance of articular cartilage surface, proteoglycan content, and chondrocyte cellularity in the medial compartment, specifically within the medial tibia (Fig. 5F–K).

### IHC for inflammatory mediators in medial tibial plateau cartilage

**Fig. 6:**
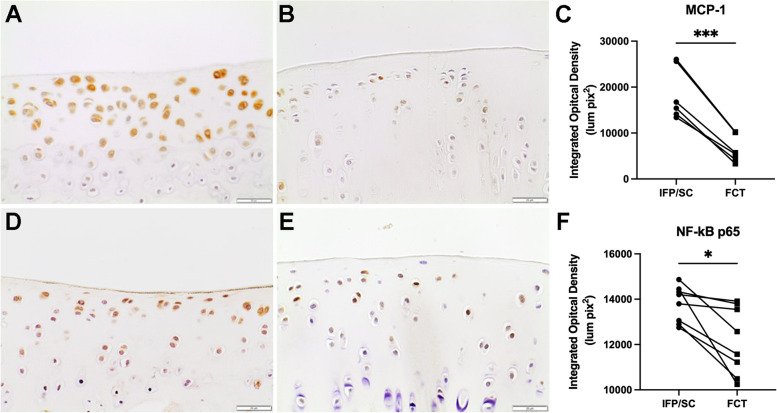
MCP-1 and NF-kB p65 immunohistochemistry of medial tibia plateau (MTP) articular cartilage. 40× objective representative images of MTP articular cartilage immunostained for MCP-1 from IFP/SC (**A**) and FCT-containing knees (**B**) of the same animal. (**C)** Figure representing quantitative analysis of MCP-1-stained tissue subtracted from IgG control tissue for all samples. IHC for NF-kB p65 was also significantly decreased in FCT knees compared (**E**) to IFP/SC knees (**D**). (**G**) Figure representing quantitative analysis of p65 stained tissue subtracted from IgG control tissue for all samples. Data was normally distributed and compared using parametric ratio paired *t* test^†^. *** *P* < 0.0005 and * *P*< 0.05

Similar to the IFP/SC and FCT protein expression, immunostaining for MCP-1 (Fig. 6A–C; *P*<0.0001) and NF-kB p65 (Fig. 6D–F; *P*=0.0127) in the medial tibial plateau showed a significant decrease in FCT-containing knees when compared to IFP/SC hind limbs.

### Biomechanical indentation results and trace elements

**Fig. 7:**
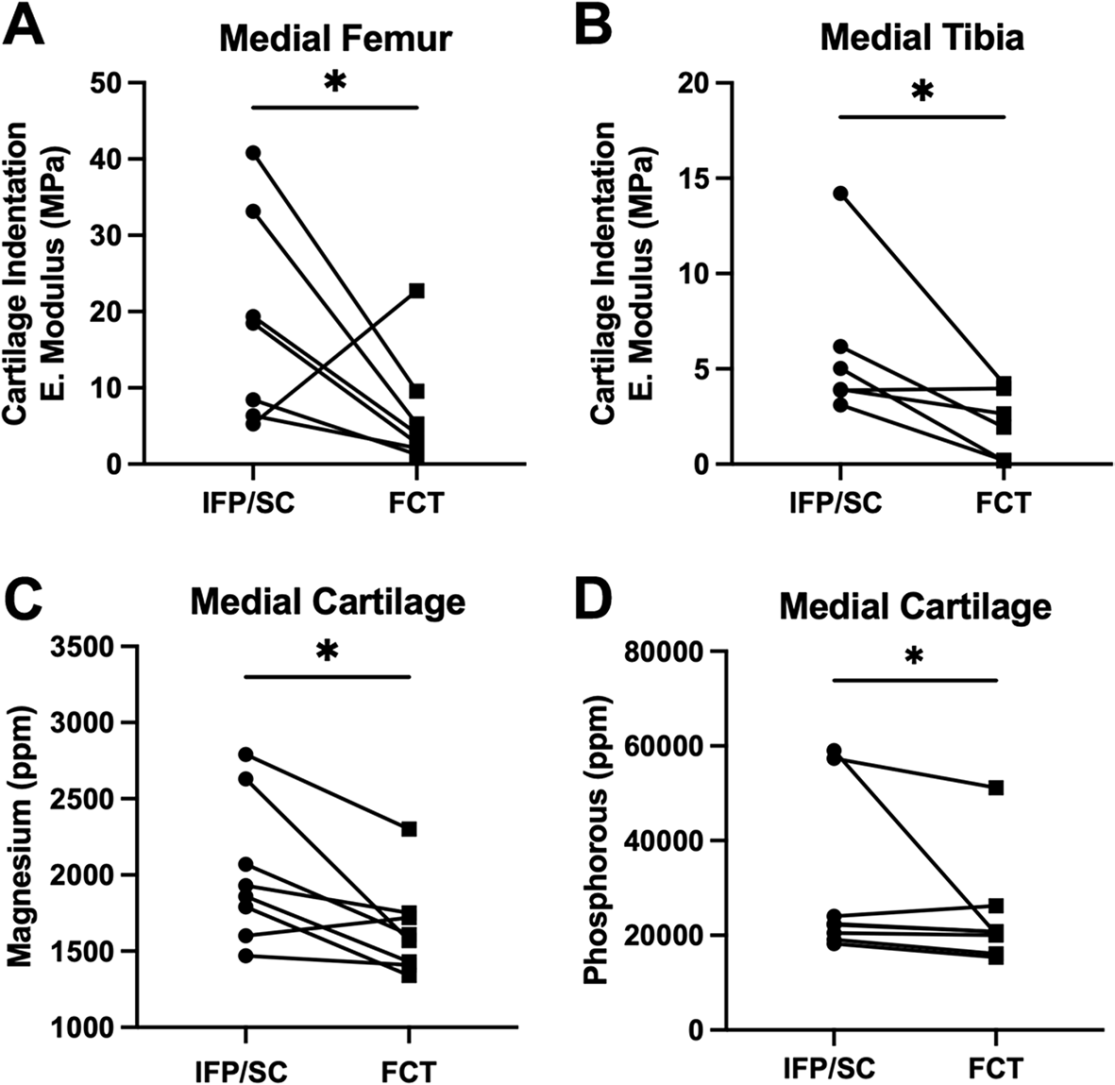
Indentation and trace element concentration of medial cartilage. Equilibrium elastic modulus of the medial femur (**A**) and medial tibia (**B**) were lower in FCT-containing knees when compared to the IFP/SC. Medial cartilage analyzed for trace element revealed a lower concentration of Mg (**C**) and P (**D**) in cartilage from FCT knees compared to cartilage collected from IFP/SC knees. Data was normally distributed and compared using parametric ratio paired *t* test^†^. * *P*< 0.05

Comparative stress-relaxation tests for cartilage revealed that the equilibrium modulus of medial hemi-joint articular cartilage was significantly decreased in FCT knees compared to the IFP/SC-containing sham controls (Fig. 7A; *P*=0.0413, Fig. 7B; *P*=0.0402). Similarly, there was a significantly lower equilibrium modulus in medial posterior menisci (Supplemental Table 8; *P*=0.0245). Medial cartilage collected from these tibiae and femora showed lower concentrations in 2 out of 5 trace elements in FCT-containing knees compared to native IFP/SC; specifically, magnesium (Mg) (Fig. 7C; *P*=0.0168) and phosphorous (P) (Fig. 7D; *P*=0.0547). Mean values (and 95% confidence intervals) for all trace elements tested in the medial and lateral cartilage can be found in Supplemental Tables 9 and 10. The lateral compartment did not have this same difference in equilibrium modulus or trace elements; notably, however, zinc (Zn) was increased within lateral cartilage collected from the IFP/SC knees when compared to the FCT hind (Supplemental Table 10; *P*=0.0078).

## Discussion

### Treadmill-based gait analyses

Mobility assessments via treadmill-gait and voluntary weight-bearing systems were utilized to identify potential alterations in gait parameters given the unilateral removal of the IFP/SC. The changes in gait over time for stride length, stance, and propel were an expected function of growth between 3 and 7 months of age [47–49]. In spite of our documented decrease in structural OA, removal of the IFP/SC compared to the limb with the native IFP/SC did not result in any changes in midline distance, stride length, swing, stance, brake, or propel time. This is perhaps unsurprising, as (1) except for the removal of the IFP/SC, each knee underwent an identical surgical procedure; (2) cranial/anterior drawer tests did not detect a significant difference between matched knees; and (3) structural joint changes are not necessarily accompanied by direct correlations to presenting clinical signs, particularly in the short-term [34, 50–54].

### Characterization of IFP versus replacement tissue

In this animal model, histopathologic examination of knee joints revealed that removal of the IFP/SC resulted in the development of a thick band of collagenous FCT in the space previously occupied by the native tissue. These microscopic findings were supported by transcript expression data, which demonstrated significantly decreased expression of key adipocyte-related molecules, ADIPOQ, LEP, and FASN. Notably, humans undergoing total knee arthroplasty combined with IFP/SC removal have shown a similar expansion/proliferation of residual tissue in the remaining void, with evidence of tissue remodeling characterized by loss of fat cells and deposition of large quantities of densely packed collagen fibers [55]. These data are also consistent with those of Kumer *et al.* [56] who accessed clinical and functional outcomes of Hoffa’s disease treated with high-portal arthroscopic resection and found that adipose tissue was replaced by fibrous tissue in chronic cases [56]. Additionally, these findings date back to the hallmark study conducted by Drs. Hoffa and Becker, which described this replacement process as hyperplasia and granulation of the remaining tissue such that it became interspersed with strong fibrous strings. Ultimately, endothelial cells were joined to fibrous tissue without any intervening fat [57].

Of note, MMP-2 (gelatinase A, type IV collagenase) is one of the major extracellular matrix degrading proteases and has been shown to breakdown basement membrane, which consists mainly of collagen type IV, laminin, and proteoglycans [58, 59]. Within the context of OA, MMP-3 and MMP-13 are traditionally investigated because of their contribution/role in the pathogenies of OA. Alternatively, in the current study, we found it interesting that MMP-2 and COL2A1 expression were increased in the FCT. This finding may reflect an inherent difference in the FCT versus native adipose tissue or may indicate that this replacement tissue was still under remodeling at the time point investigated. It would be worthwhile to include investigations pertinent to additional MMPs, including MMP-3 and MMP-13, and collagen types, such as collagen types 1 and 3, in continued work.

### Characterization of joint pathology

Importantly, the current study revealed that early removal of the IFP/SC prior to disease onset in an animal model of primary OA appears to have short-term benefits. Specifically, clinical microCT and histologic OA scores were worse in the knee containing native adipose tissue relative to the knee with the replacement FCT. To the authors’ knowledge, previous research in animal models of primary OA have not reported the influence of IFP/SC removal on disease pathogenesis. Collins *et al*. [60], however, utilized a murine model of lipodystrophy that examined the direct contribution of adipose tissue in an injury model of post-traumatic osteoarthritis (PTOA) induced by destabilization of the medial meniscus (DMM). Their findings indicated that adipose tissue itself may promote PTOA susceptibility directly through adipokine signaling, triggering systemic inflammation that localizes within the joint. However, the direct mechanism in the case of PTOA remains to be determined [61]. While not analogous to our current work, as these clinical projects focused on the removal of the IFP/SC at end-stage OA, it should also be mentioned that numerous studies have investigated the effects of IFP excision in total knee arthroplasty/replacement (TKA/TKR) as it relates to Knee Society Score [53, 54, 62, 63]. This scoring system is composed of five components: patient demographics, objective knee score (completed by the surgeon), patient expectations, patient satisfaction score, and, lastly, functional knee score (completed by the patient) [63]. To date, these studies have reported inconclusive/discrepant results in regards to either the benefit or drawback of IFP/SC removal; recent reviews of the effect of IFP excision in TKA found no difference in anterior knee pain, range of motion, or function in the patient with OA [55, 56, 63–65]. Thus, our project is the first study to focus on the potential benefit of IFP/SC removal on OA pathogenesis as a preventive therapy.

In this regard, it is necessary to consider both the inflammatory and/or biomechanical benefit of the FCT versus the native IFP/SC. In terms of the inflammatory contribution of the IFP/SC to OA, Clockaerts *et al.* [7] and others [64, 65] have reported that this adipose depot, particularly in cases of obesity, can cumulatively secrete cytokines, interferons, adipokines such as fatty acid binding protein 4 (FABP4) [66–68], and growth factors, all of which exert local signaling effects on articular cartilage and synovial cells [7, 64, 65]. Of interest, studies have reported that individual cellular components of the IFP/SC may contribute to OA. First, this depot serves as both a site of inflammatory/immune cell infiltration, which can provide an origin of pro-inflammatory cytokines and MMP expression. These migrating cells, including macrophages and lymphocytes, also interact and influence resident adipocytes. Second, adipocytes, themselves, are capable of secreting certain molecular markers and products able to initiate a local inflammatory response [64, 65].

Evidence to support the diverging functional characteristics and/or inflammatory nature of the IFP/SC versus FCT in the current work was provided by transcript expression data and confirmatory IHC findings. As mentioned above, it is established that elevated pro-inflammatory cytokines in OA joints play a role in cartilage homeostasis [69–72]. In particular, studies have shown that NF-κB participates in many OA-associated changes, including chondrocyte catabolism, chondrocyte survival, and synovial inflammation [73]. Specifically, NF-κB signaling pathways mediate critical events in the inflammatory response by promoting transcription of genes encoding for cytokines and stimulating production of MMPs by synoviocytes, macrophages cells, or chondrocytes [5, 74]. Notably, injury-induced cartilage lesions were alleviated by the knockdown of this mediator by specific small interfering RNA in animal models [75, 76]. Here, we demonstrated that the FCT downregulated NF-κB and its related nuclear orphan receptor, NR4A2, as well as NF-κB regulated genes, MCP-1, CAT, and C3. Protein expression further confirmed the local decrease of one key subunit of NF-κB and MCP-1 expression within the FCT. We postulate a reduction of these inflammatory mediators may have led to decreased OA changes; indeed, MCP-1 and NF-κB p65 protein expression was also decreased in the medial tibial cartilage associated with the FCT.

In addition to the changes observed between the FCT, IFP/SC, and articular cartilage, clinical microCT scoring revealed that FCT-containing knees had decreased numbers, size, and volume of peri-articular osteophytes compared to joints with the native IFP/SC. Osteophytes are bony outgrowths arising from the periosteum of joint margins and typically increase with OA disease progression [77]. They are thought to provide added stability to joints with OA [78], although other functions may exist. Of note, osteophytes are commonly associated with specific growth factors and extracellular matrix molecules. For example, a rat model of OA revealed that exogenous leptin treatment resulted in metabolic dysregulation of chondrocytes. The level of leptin expression was related to osteophytes and OA cartilage of rats treated with this particular adipokine [79]. Indeed, the potential connection between leptin and osteophyte formation has not been heavily explored, and interactions between IFP/SC and bone warrant further investigation.

From a biomechanical standpoint, medial cartilage indentation from the tibia and femur provided higher equilibrium modulus from knees containing the IFP/SC when compared to FCT. Previous research in human cartilage samples has reported indentation measurements of equilibrium modulus with values from 0.020 ± 0.003 MPa at the cartilage superficial zone to 6.44 ± 1.002 MPa at the calcified layer [80]. Such differences in biomechanical properties of aging cartilage are of particular interest because the mismatch of mechanical properties between contralateral knees could provide different strain profiles, potential alteration of congruency, and redistribution of stress across the joint surface [80, 81]. Furthermore, the observed higher concentrations of trace elements Mg and P in articular cartilage from knees containing native IFP/SC could be reflective of pathologic changes such as calcification. Indeed, it was curious that Mg values in the IFP/SC and FCT themselves were not significantly different but other joint tissues were. This raises the question as to how joint tissue may be communicating and/or regulating the mineral content of articular cartilage. Mg and P concentrations vary depending on biological, dietary, and environmental factors. Mg plays a key role in bone mineral homeostasis and bone cell formation. Stored Mg contributes to bone stabilization and influences the osteoblasts and osteoclasts needed for bone growth and repair [82]. Elevated Mg cartilage concentrations have been reported to have a negative effect on bone stiffness and strength. Further, excessive P intake can lead to increased loss of bone mass and deterioration of biomechanical properties [83]. Further work is warranted to tease out the relevance of these findings to articular cartilage health and overall joint homeostasis.

The relative increase in subchondral trabecular bone vBMD of medial tibiae and femurs associated with the native IFP/SC was an interesting finding, which was supported by the following triad of data: significantly increased Mg, coupled with a significant decrease in P, and a trending increase in calcium. While exact values for this parameter are challenging to compare among available manuscripts due to protocol differences, some observations consistent with our findings are worthy of mention. Specific to work performed in guinea pigs, Wang *et al.* [84] demonstrated that BMD increased as Hartley guinea pigs aged from 1 to 3 months and began developing pathology. Further, while this difference was only present in the lateral compartment between strains, there was also a significant increase in BMD in Hartley guinea pigs compared to a control strain [84]. Of note, much evidence exists to support a positive association between long bone BMD and radiographic OA; however, these findings are not consistent [85]. Thus, while the mechanisms remain to be defined, it is noteworthy that removal of the IFP/SC may have promoted a vBMD more conducive for maintenance of healthy articular cartilage.

Considerations that should be noted in regards to this study include the fact that the current assessment involves only male animals; continued work will address findings in female guinea pigs. Furthermore, removal of the IFP/SC occurred prior to OA onset may have limited clinical application for the typical patient with OA. Indeed, examining the effects of IFP/SC removal at additional time points in the course of knee OA progression is needed to dissect the long-term benefit of IFP/SC removal on knee health. From a mechanical perspective, future work is warranted to directly measure the biological elastic content of the IFP/SC to include testing on the properties of the tissue and components of its extracellular matrix. Further, it is not known whether the anti-inflammatory benefits and decreased OA outcomes seen in the present work will hold true in a longer-term scenario. Finally, we acknowledge that our study design utilized the contralateral knee as a sham control to focus on within animal contrasts and comparison. It may be important to examine bi-lateral IFP/SC removal and/or a contralateral naïve control limb to account for compensatory limb considerations. In spite of this, we have achieved a noteworthy delay of OA onset and/or progression in the Hartley guinea pig model.

## Conclusions

This study demonstrates that removal of the IFP/SC reduced local inflammation, altered biomechanical properties of medial articular cartilage when compared to the knee containing native adipose tissue. This resulted in short-term improved joint pathology associated with OA in male Hartley guinea pigs.

## Supplementary Information

**Additional file 1.** Supplementary material.

## Data Availability

The data that support the findings of this study are available for the corresponding author upon reasonable request.
