# Supplementary material for: Early removal of the infrapatellar fat pad/synovium complex beneficially alters the pathogenesis of moderate stage idiopathic knee osteoarthritis in male Dunkin Hartley guinea pigs
Source: Arthritis Res Ther. 2022 Dec 28;24:282. doi: 10.1186/s13075-022-02971-y (PMC9795160; doi:10.1186/s13075-022-02971-y)
Supplement: Supplementary file 1 — Additional file 1. Supplementary material. [file 13075_2022_2971_MOESM1_ESM.zip › Supplemental Table 1. Nanostring Accession .pdf]

Supplemental Table 2. Primer sequences used for Nanostring gene expression analysis. The length of all target sequences are 99 base pairs.

Gene	Accession Number	Target Sequence
ADIPOQ	XM_003476936.2	CTCCCTCTAAAAATCACATAGTACATTGCCAGCCCTAAAGAAGACCTGGTCCCTCCAGGAGCTCTATTGG ACAGTAGTAAAATTTGCTGATGGATTTCAC
ACTB	NM_01172909.1	TGCTTCTAGGCGGACTGTTACTACTTTGCTGCGTTACACCCTTTCTTGACAAAAAAACCTAACTTGCGCG CAGAAAACGAGATGAGATTGGCATGGCTTT
C3	NM_001172903.1	CCCGAGTTGGTCAACATGGGGCAGTGGAAAATCCAGGCCTTCTACGAGAACTCGCCCAAGCAAGTCTTC TCGGCTGAGTTCGAGGTGAAAGAATACGTGC
CAT	NM_001172925.1	CCGAGGTCCCCTGTCACGTTCTGAGGCTATTGTCGGACACCATGGCGGACAGCCGAGATCCAGCCAGC GACCAGATGAAGCACTGGAAGGAGGAGCGGG
COL2A1	XM_005006506.3	TTGTGGGGACAGAGGCCGTCTCTGCGCCCCGCCCGATCAGGCACTGGGCACTAGGTGGGGGCGGGAG CAGGGTTCTCGGGAGGGGGGGTCCGGTCCAGG
FASN	XM_013147192.1	ACAGCATGGTGGGCTGCCAGCGTGCAATGATGGCCAACCGCATTTCCTTCTTCTTTGACTTCAAAGGGC CCAGCATCGCCCTGGACACAGCCTGCTCCTC
GAPDH	NM_001172951.1	AGAGCTGAATGGGAAGCTCACAGGTATGGCCTTCCGTGTACCCACACCTAATGTGTCGGTTGTGGATCT GACCTGCCGCCTGGAGAAACCGGCCAAATAC
LEP	XM_003475050.2	CCATGCAAAAACTCCAGGATGATGCCAAGTTCCTCATCAAGACCATTATCAACAGGATCCGTAACATTTC ACACCTGCAGTCGGTGTCCTCTAAACACAA
MCP-1	NM_001172926.1	AGTGGGTGCAGGACTACATTGCCAAACTGGACCAGAGAACCCAGCAGAAACAGAACTCAACTGCACCTC AAACTTCAAAGCCTTTGAACATTCGTTTCAC
MMP-2	XM_003477541.3	GGAGATCTGCAAACAGGACATCGTCTTTGATGGCATCTCTCAGATCCGTGGTGAGATCTTCTTCTTCAAG GACCGGTTCATTTGGAGAACAGTGACACCA
NF-kB 1	XM_003468027.3	GCACCGGACCAGGGTATGGCTTCCCACACTACGGATTCCCGACATACAGTGGGATCGCCTTCCATCCTG GAACCACCAAGTCCAACGCCGGCATGAAACA
NF-kB p65	XM_003468074.3	GCGAACCCATGGAGTTCCAGTACCTTCCAGACACAGATGATCGTCACCGGATTGAGGAGAAGCGCAAAA GGACATATGAGACCTTCAAGAGCATCATGA
NR4A2	XM_003477541.3	GCTACAGTTACCACTCTTCGGGAGAATACAGCTCCGATTTCTTAACTCCAGAGTTTGTCAAGTTTAGCAT GGACCTCACCAACACTGAAATCACTGCCAC
SDHα	DQ402978.1	ACTGCTGCATACCTTGTATGGCAGGTCTCTGCGATATGATACCAGCTATTTTGTAGAGTATTTTGCCTTAG ATCTCCTGATGGAAAATGGGGAGTGCCGT
