# Supplementary material for: Early removal of the infrapatellar fat pad/synovium complex beneficially alters the pathogenesis of moderate stage idiopathic knee osteoarthritis in male Dunkin Hartley guinea pigs
Source: Arthritis Res Ther. 2022 Dec 28;24:282. doi: 10.1186/s13075-022-02971-y (PMC9795160; doi:10.1186/s13075-022-02971-y)
Supplement: Supplementary file 1 — Additional file 1. Supplementary material. [file 13075_2022_2971_MOESM1_ESM.zip › Supplemental Table 2. Weight bearing parameters_ESM.pdf]

Supplemental Table 2. Additional voluntary weight bearing parameters (Tekscan Rodent Walkway System). Values are represented as Mean, 95% CI interval upper and lower limits. P-values bolded are statistically significant. P-values represent significance of Two-Way ANOVA (or Mixed Model) with a Tukey post hoc test analysis.

	**Left Hind (sham)**	**Right Hind (IFP Removal)**	**Left vs Right Hind (P-value)**	**Time (P -value)**
**Maximum Force (%BW)**	35.41 [27.74,43.07]	32.39 [24.19,40.59]	0.2319	******0.0001***
**Stride Velocity (cm/sec)**	42.56 [33.65,51.48]	42.73 [33.24,52.21]	0.9706	****0.0296***
**Maximum Peak Pressure (kPA)**	51.83 [45.63, 59.50]	47.81 [39.00, 58.63]	0.1160	*****0.0058***
**Maximum Force (kg)**	0.267 [0.22, 0.31]	0.258 [0.21,0.30]	0.8220	*******<0.0001***
**Force Time Integral [FTI (%BW*sec)]**	6.602 [5.00, 8.35]	6.686 [5.13, 9.55]	0.9205	0.2457
**Force Time Integral [FTI (kg*sec)]**	0.570 [0.03, 0.08]	0.059 [0.03, 0.11]	0.8472	****0.0163***
**Stride Length (cm)**	13.93 [11.90, 15.48]	13.79 [11.59, 16.74]	0.7123	*****0.0012***
**Stride Time (sec)**	0.431 [0.32, 0.54]	0.421 [0.30, 0.56]	0.7918	****0.0240***
**Stance Time (sec)**	0.344 [0.22, 0.50]	0.331 [0.20, 0.46]	0.7447	******0.0004***
**Swing Time (sec)**	0.126 [0.11, 0.14]	0.114 [0.09, 0.13]	0.3571	0.5134
