# Supplementary material for: Early removal of the infrapatellar fat pad/synovium complex beneficially alters the pathogenesis of moderate stage idiopathic knee osteoarthritis in male Dunkin Hartley guinea pigs
Source: Arthritis Res Ther. 2022 Dec 28;24:282. doi: 10.1186/s13075-022-02971-y (PMC9795160; doi:10.1186/s13075-022-02971-y)
Supplement: Supplementary file 1 — Additional file 1. Supplementary material. [file 13075_2022_2971_MOESM1_ESM.zip › Supplemental Table 3. Biomechanic Tests IFP vs FCT_ESM.pdf]

Supplemental Table 3. Biomechanical Analyses. Mean values (with 95% confidence interval) for Biomechanic measurements for IFP/SC vs FCT limbs. Normally distributed data with similar variance were compared using parametric ratio t tests†. Data with non-Gaussian distribution were compared using non-parametric Wilcoxon matched – pairs signed rank test ˣ.

		IFP/SC (sham)	FCT (IFP removal)	P-value
**Cranial/Anterior Drawer**		5.57<br>[3.56,7.58]	4.66<br>[4.16,5.16]	0.6875ˣ
**Patellar Tendon**	Width	3.43<br>[2.90,3.95]	3.50<br>[3.02,3.98]	0.7929†
	Thickness	1.72<br>[1.33, 2.10]	1.77<br>[1.52,2.02]	0.8438ˣ
**Pull to Failure**	Cross-sectional Area (mm^2)	18.12<br>[14.75,21.49]	19.15<br>[15.65,23.37]	0.6511†
	Elastic Modulus (MPa)	29.33<br>[4.36,54.31]	23.89<br>[12.42,35.37]	0.5781ˣ
	Ultimate Load (N)	107.60<br>[78.83,136.30]	125.60<br>[103.1,148.1]	0.2989†
	Ultimate Stress	6.371<br>[4.17,8.57]	6.833<br>[4.83,8.84]	0.6879†
	Ultimate Strain	0.46<br>[0.32,0.60]	0.50<br>[0.26,0.74]	0.9646†

Methods: Cranial/anterior drawer was conducted on whole tibiofemoral joints to quantitate the total cranial displacement of the tibia relative to the femur. Whole joints were potted in cement, mounted in cylindrical holding tubes and mounted with the knee flexed at 90° in a servo-hydraulic testing system (MTS, Eden Prairie, Minnesota) with an 8.9 N load cell. A 2-3 N preload was applied to the knee joint before a displacement of 1mm in 1 sec was applied to distract the joint. Load, displacement and time were recorded at 1024 Hz. Following cranial drawer tests, the joint was disarticulated and the patella, patellar tendon, associated underlying structures (IFP/SC or FCT), and tibia were isolated for pull to failure tests to determine the material properties of the tendon and underlying IFP/SC or FCT. The tibia remained potted and attached to the material testing system. Cross-sectional area of the patellar tendon was measured using vernier calipers. The patella was then mounted in an upper fixture such that it was encased in bone cement to ensure no slippage of sample. Following a 1 N preload, the patellar tendon unit was then pulled in extension at 10%-gauge length/second until failure. Cross-sectional area, ultimate load, ultimate stress, ultimate strain and elastic modulus were determined. Elastic modulus was taken as the slope of the linear portion of the stress-strain curve.
