# Supplementary material for: Early removal of the infrapatellar fat pad/synovium complex beneficially alters the pathogenesis of moderate stage idiopathic knee osteoarthritis in male Dunkin Hartley guinea pigs
Source: Arthritis Res Ther. 2022 Dec 28;24:282. doi: 10.1186/s13075-022-02971-y (PMC9795160; doi:10.1186/s13075-022-02971-y)
Supplement: Supplementary file 1 — Additional file 1. Supplementary material. [file 13075_2022_2971_MOESM1_ESM.zip › Supplemental Table 4. Complete custom gene panel.pdf]

Supplemental Table 4. Complete Custom Guinea Pig Nanostring Gene Panel. Mean values (with 95% confidence interval) for normalized mRNA counts for IFP/SC vs FCT limbs. Normally distributed data with similar variance were compared using parametric ratio t tests†. Data with non-Gaussian distribution were compared using non-parametric Wilcoxon matched – pairs signed rank test×. Normalized mRNA counts less than detectable limits (BDL = below detectable limit)

Gene	Accession number	IFP/SC	FCT	P-value
ACAN	XM_013158932.2	12.45 [4.94,19.95]	18.18 [11.70,24.67]	0.0944†
ADIPOQ	XM_003476936.2	184.30 [73.88, 294.7]	54.56 [26.38, 82.75]	*****0.0039×***
AKT	XM_003463112.3	167.70 [86.98, 248.40]	175.70 [100.40,251.00]	0.7344×
BAD	XM_013150725.2	14.36 [8.65, 20.08]	21.84 [8.44, 35.25]	0.5703×
BAK	XM_013156999.2	16.02 [8.87, 23.17]	18.25 [7.92, 28.78]	0.5703×
BAX	XM_003465527.4	25.78 [19.43, 32.13]	32.41 [23.22, 41.60]	0.1289×
BCL-2	XM_003474076.3	25.13 [14.57,35.68]	29.70 [19.99, 39.41]	0.4258×
BECN1	XM_005002460.3	90.95 [57.56, 124.30]	88.02 [66.93, 109.10]	>0.9999×
BIM	XM_013147388.2	18.50 [14.13, 22.86]	18.88 [13.53, 24.24]	>0.9999×
BMP-7	XM_003463644.4	*BDL*	*BDL*	*BDL*
C3	NM_001172903.1	34.42 [22.60, 43.80]	14.89 [8.078, 20.44]	****0.0195×***
CASP1	XM_013155657.1	12.30 [9.236, 15.36]	15.15 [11.30, 19.00]	0.2787†
CASP3	XM_003469171.4	*BDL*	*BDL*	*BDL*
CASP8	XM_013145226.2	*BDL*	*BDL*	*BDL*
CASP9	XM_003471180.4	7.430 [3.964, 10.90]	9.766 [2.623, 16.91]	0.6523×
CAT	NM_001172925.1	74.27 [55.71, 92.84]	62.82 [49.28, 76.35]	****0.0131†***
CBS	XM_003463923.4	*BDL*	*BDL*	*BDL*
CCL-2	NM_001172926.1	85.11 [39.52, 130.70]	41.91 [25.81, 58.00]	****0.0117×***
CD163	XM_005003802.2	32.15 [14.51, 49.79]	29.00 [19.16, 38.84]	0.9102×
CGRP	NM_001172933.1	*BDL*	*BDL*	*BDL*
CISH	XM_003476702.3	10.07 [6.95, 13.20]	9.198 [4.77,13.64]	0.36721†
COL10A1	XM_013148983.1	5.97 [3.33, 8.61]	14.05 [1.25, 26.84]	0.1289×
COL2A1	XM_005006506.3	13.79 [8.32, 19.25]	50.88 [12.68, 89.07]	****0.0178†***
CUL3	XM_003463982.3	24.41 [5.55, 43.28]	19.05 [14.60, 23.50]	0.8203×
Cidec	XM_023562573.1	9.90 [5.86, 13.95]	7.43 [4.62, 10.25]	0.1538†

Csnk2a1	XM_003476763.4	48.97 [32.51, 65.44]	58.95 [40.02, 77.88]	0.3594$^{\times}$
FGF-18	XM_003473332.4	103.4 [53.82, 153.00]	137.0 [74.98, 198.90]	0.4785†
FTH-1	NM_001172847.1	765.30 [330.50, 1200]	666.5 [346.80, 986.20]	0.9102$^{\times}$
Fasn	XM_013147192.1	33.96 [22.15, 45.78]	21.48 [15.69, 27.27]	*0.0130†
GPS2	XM_003466264.4	9.32 [6.37, 12.27]	17.42 [9.18, 25.66]	*0.0489†
GPx	XM_003476448.4	1060 [528.2, 1592]	1107 [771.4, 1442]	0.5703$^{\times}$
GPx4	NM_001256319.1	20.53 [13.18, 27.87]	28.50 [16.54, 40.46]	0.3008$^{\times}$
GSK3b	XM_003469225.4	10.13 [7.51, 12.75]	15.45 [7.18, 23.71]	0.2617$^{\times}$
Gap43	XM_013151711.2	15.14 [4.91, 25.38]	10.23 [5.26, 15.20]	0.6523$^{\times}$
HAMP	XM_003466576.3	9.157 [6.60, 11.71]	11.16 [7.97, 14.36]	0.4358$^{\times}$
HIF1-a	XM_013155206.1	9.64 [4.75, 14.53]	9.71 [5.52, 13.90]	0.5703$^{\times}$
HMGB1	XM_013141181.2	33.29 [16.53, 50.05]	38.49 [24.97,52.01]	0.4961$^{\times}$
HMOX-1	XM_003462326.3	55.11 [29.48, 80.75]	65.14 [50.46,79.83]	0.2240†
IFN-g	NM_001172874.1	*BDL*	*BDL*	*BDL*
IL-10	NM_001260485.1	*BDL*	*BDL*	*BDL*
IL-1b	NM_001172968.1	*BDL*	*BDL*	*BDL*
IL-4	NM_001257263.1	*BDL*	*BDL*	*BDL*
IL-5	NM_001172970.1	*BDL*	*BDL*	*BDL*
IL-6	XM_013152399.1	*BDL*	*BDL*	*BDL*
JAK2	XM_003472261.4	6.29 [2.15, 10.41]	6.36 [3.14, 9.59]	0.9102$^{\times}$
KEAP1	XM_003460852.3	209.2 [141.6, 276.9]	221.7 [160.7, 282.8]	0.6047†
LEP	XM_003475050.2	26.05 [17.02, 35.08]	14.19 [8.24, 20.14]	***0.0005†
MAPK	XM_003478061.3	28.08 [23.09, 33.07]	33.51 [22.61, 44.41]	0.4598†
MAPK3	XM_003478227.4	13.14 [9.15, 17.13]	18.21 [10.82, 25.59]	0.2825†
MCL1	XM_023561171.1	6.37 [4.07, 8.68]	8.59 [3.15, 14.03]	0.8828$^{\times}$
MMP-2	XM_003477541.3	78.18 [43.59, 112.8]	203.9 [132, 275]	**0.0018†
MMP-3	XM_003472809.3	*BDL*	*BDL*	*BDL*
MMP-9	XM_003467793.2	*BDL*	*BDL*	*BDL*

MMP-13	XM_003472818.2	*BDL*	*BDL*	*BDL*
mTOR	XM_003471204.3	19.43 [10.75, 28.11]	21.80 [12.04, 31.57]	0.8568†
MAPK14	XM_023563297.1	20.86 [15.03, 26.70]	25.15 [16.79, 33.50]	0.3353†
NFE-2	XM_003476239.2	*BDL*	*BDL*	*BDL*
NF-kB p50	XM_003468027.3	53.64 [33.92, 73.36]	30.21 [21.84, 38.58]	*0.0117$^{\times}$
NFE2L2	XM_003478542.4	13.16 [4.90, 21.42]	12.41 [9.20, 15.62]	>0.9999$^{\times}$
NGF	XM_023561339.1	9.38 [2.72, 1605]	11.03 [4.57, 17.49]	0.8203$^{\times}$
NOS1	XM_013141712.2	*BDL*	*BDL*	*BDL*
NOS2	NM_001172984.1	*BDL*	*BDL*	*BDL*
NOS3	NM_001172985.1	*BDL*	*BDL*	*BDL*
NURR1	XM_003478688.4	16.35 [6.39, 26.30]	8.68 [3.25,14.11]	*0.0273$^{\times}$
Nrf1	XM_013158203.2	10.47 [7.691, 13.24]	17.21 [11.57, 22.85]	0.0547$^{\times}$
NQO1	NM_001172986.1	*BDL*	*BDL*	*BDL*
PCSK9	XM_023562560.1	*BDL*	*BDL*	*BDL*
PPARG	XM_013144495.1	*BDL*	*BDL*	*BDL*
PRDX1	XM_013144242.1	27.88 [20.47,35.29]	42.25 [27.92,56.58]	0.0870†
PTGS-1/COX-1	XM_003470662.3	8.13 [3.28, 12.97]	11.23 [4.87, 17.58]	0.2756†
PTGS-2/COX-2	NM_001173007.1	*BDL*	*BDL*	*BDL*
Pik3r1	XM_003462679.4	22.95 [15.53, 30.36]	31.55 [16.53, 46.57]	0.4101†
Protein Kinease C Zeta	XM_013153031.1	6.73 [3.07, 10.39]	8.03 [5.84, 10.22]	0.5703$^{\times}$
RIPK1	XM_003468780.4	11.76 [7.74, 15.79]	17.69 [6.71, 28.66]	0.3594$^{\times}$
RUNX2	XM_013156585.1	*BDL*	*BDL*	*BDL*
SESN2	XM_003471268.4	9.917 [6.07, 13.77]	9.81 [6.82, 12.80]	0.8863†
SLC39A14/ZIP14	XM_005008109.2	*BDL*	*BDL*	*BDL*
SLC7A11	XM_003476798.4	*BDL*	*BDL*	*BDL*
SLC40A1/FPN1	XM_005008713.2	12.08 [8.79, 15.36]	16.23 [10.59, 21.88]	0.1983†
SOCS3	XM_013147101.2	53.65 [13.51, 93.79]	36.44 [25.12, 47.75]	0.6523$^{\times}$
SOD-1	XM_003467248.3	28.78 [21.66, 35.91]	40.28 [26.13, 54.43]	0.1320†

SOD-2	XM_003466367.3	32.08<br>[9.56, 54.60]	31.01<br>[25.61, 36.41]	0.4258$^{\times}$
SOD-3	XM_003467399.3	43.85<br>[29.48, 58.22]	44.01<br>[26.78, 61.24]	>0.9999$^{\times}$
Substance P	NM_001172899.1	*BDL*	*BDL*	*BDL*
STAT1	XM_003478777.4	23.20<br>[17.60, 29.01]	26.24<br>[20.21, 32.28]	0.3550†
STAT2	XM_005006399.3	22.67<br>[12.81, 32.53]	21.32<br>[14.44, 28.20]	0.9544†
STEAP4	XM_003474977.4	8.021<br>[5.82, 10.22]	10.57<br>[6.53, 14.61]	0.3655†
Serpine1	XM_003469906.3	43.96<br>[18.26, 69.67]	43.92<br>[30.59, 57.24]	0.5492†
TFRC	NM_001251822.1	11.78<br>[6.12, 17.44]	15.34<br>[7.69, 23.00]	0.8203$^{\times}$
TGF-β	NM_001173023.1	25.33<br>[10.53, 40.14]	23.46<br>[15.43, 31.50]	0.8203$^{\times}$
TIMP-1	XM_005000239.2	15.84<br>[1.25, 30.42]	13.21<br>[6.61,19.82]	>0.9999$^{\times}$
TIMP-2	NM_001173024.1	15.45<br>[9.30, 21.60]	19.69<br>[12.46, 26.93]	0.4424†
TNF	NM_001173025.1	*BDL*	*BDL*	*BDL*
TRPA1	NM_001198770.1	8.103<br>[3.71, 12.50]	12.48<br>[2.22, 22.74]	0.9102$^{\times}$
TRPV 1	NM_001172652.1	*BDL*	*BDL*	*BDL*
TRPV 4	XM_023560824.1	*BDL*	*BDL*	*BDL*
TXN	XM_003463781.4	8.83<br>[1.64, 16.02]	15.10<br>[6.10, 24.10]	0.2500$^{\times}$
ULK1	XM_004999396.3	12.02<br>[4.82, 19.23]	10.85<br>[6.12,15.57]	0.6406$^{\times}$
VGF	XM_013152538.2	*BDL*	*BDL*	*BDL*
WNT	XM_013159109.1	*BDL*	*BDL*	*BDL*
b-CAT	XM_003477001.3	74.38<br>[46.30,102.50]	83.42<br>[62.38,104.50]	0.3432†
p65 (RELA)	XM_003468074.4	66.93<br>[45.28, 88.57]	45.60<br>[27.63, 63.57]	**0.0021†
