# Supplementary material for: Early removal of the infrapatellar fat pad/synovium complex beneficially alters the pathogenesis of moderate stage idiopathic knee osteoarthritis in male Dunkin Hartley guinea pigs
Source: Arthritis Res Ther. 2022 Dec 28;24:282. doi: 10.1186/s13075-022-02971-y (PMC9795160; doi:10.1186/s13075-022-02971-y)
Supplement: Supplementary file 1 — Additional file 1. Supplementary material. [file 13075_2022_2971_MOESM1_ESM.zip › Supplemental Table 5. Trace elements IFP vs FCT_ESM.pdf]

Supplemental Table 5. Atomic Absorption Spectroscopy (AAS) trace element concentrations of IFP/SC and FCT. Mean values (with 95% confidence interval) for IFP/SC and FCT collected from IFP/SC vs FCT limbs. Normally distributed data with similar variance were compared using parametric ratio t tests†. Data with non-Gaussian distribution were compared using non-parametric Wilcoxon matched – pairs signed rank test ˣ.

	Concentration (ppm)		
Trace Elements	IFP/SC (sham)	FCT (IFP removal)	P-value
**Calcium** (Ca)	3750<br>[98.50, 15100]	419<br>[275, 567]	0.8125 ˣ
**Magnesium** (Mg)	147.20<br>[32.20, 78.90]	141.60<br>[78.80, 271]	0.5756†
**Zinc** (Zn)	52.90<br>[22.80, 74.90]	276.1<br>[48.30, 1101]	0.1875†
**Iron** (Fe)	69.60<br>[20.30, 209]	82.14<br>[76.20, 120]	0.4375 ˣ
**Phosphorous** (P)	7,728<br>[1870, 16611]	11,330<br>[100, 18600]	0.0553†
