# Supplementary material for: Early removal of the infrapatellar fat pad/synovium complex beneficially alters the pathogenesis of moderate stage idiopathic knee osteoarthritis in male Dunkin Hartley guinea pigs
Source: Arthritis Res Ther. 2022 Dec 28;24:282. doi: 10.1186/s13075-022-02971-y (PMC9795160; doi:10.1186/s13075-022-02971-y)
Supplement: Supplementary file 1 — Additional file 1. Supplementary material. [file 13075_2022_2971_MOESM1_ESM.zip › Supplemental Table 6. Quanitative microCT_ESM.pdf]

Supplemental Table 6. Quantitative MicroCT. Mean values (with 95% confidence interval) for quantitative microCT measurements for IFP/SC vs FCT limbs. Values are derived from the medial and lateral tibial and femoral subchondral trabecular bone. Normally distributed data with similar variance were compared using parametric ratio t tests†. Data with non-Gaussian distribution were compared using non-parametric Wilcoxon matched – pairs signed rank test ˣ.
MT, medial tibia; LT, lateral tibia; MF, medial femur; LF, lateral femur

		**IFP/SC**	**FCT**	**P-value**
**Trabecular vBMD (g/cm^3)**	**MT**	0.47 [0.42,0.51]	0.40 [0.35,0.46]	**** 0.0483†***
	**LT**	0.49 [0.44,0.54]	0.49 [0.39,0.61]	0.9343†
	**MF**	0.39 [0.33,0.44]	0.34 [0.27,0.39]	**** 0.0297†***
	**LF**	0.27 [0.22,0.32]	0.31 [0.19,0.53]	0.5469ˣ
**Trabecular Bone Volume/Total Volume (BV/TV%)**	**MT**	29.95 [26.69,33.22]	27.47 [25.37,29.58]	0.1559†
	**LT**	28.80 [23.62,33.99]	31.36 [27.53,35.19]	0.4550†
	**MF**	29.01 [24.04,33.98]	24.73 [21.55,27.91]	0.1484†
	**LF**	19.57 [14.01,25.12]	20.92 [16.38,25.46]	0.3828ˣ
**Trabecular Number (Tb.N)**	**MT**	2.31 [2.15,2.46]	2.23 [2.09,2.37]	0.3006†
	**LT**	2.48 [2.12,2.85]	2.47 [2.24,2.71]	0.9753†
	**MF**	2.12 [1.793,2.45]	1.92 [1.67,2.17]	0.3125†
	**LF**	1.78 [1.48,2.07]	1.78 [1.53,2.04]	0.3828ˣ
**Trabecular Thickness (mm)**	**MT**	0.13 [0.12,0.14]	0.12 [0.12,0.13]	0.3786†
	**LT**	0.13 [0.10,0.16]	0.13 [0.12,0.14]	0.7422†
	**MF**	0.14 [0.13,0.14]	0.13 [0.12,0.14]	0.1094†
	**LF**	0.11 [0.09,0.12]	0.12 [0.11,0.13]	0.2500ˣ
**Trabecular Space (Tb.SP)**	**MT**	0.29 [0.27,0.32]	0.29 [0.27,0.31]	0.5306†
	**LT**	0.24 [0.19,0.29]	0.25 [0.22,0.28]	>0.9999†
	**MF**	0.31 [0.27,0.34]	0.33 [0.31,0.36]	0.3125†
	**LF**	0.33 [0.29,0.36]	0.32 [0.30,0.34]	0.7439ˣ
**Cortical vBMD (g/cm^3)**	**MT**	0.85 [0.79,0.90]	0.86 [0.82,0.90]	0.6785†
	**LT**	0.87 [0.82,0.91]	0.86 [0.84,0.89]	0.8143†
	**MF**	0.84 [0.70,0.98]	0.91 [0.86,0.95]	0.6406†
	**LF**	0.98 [0.94,1.01]	0.92 [0.82,1.02]	0.3828ˣ
**Cortical Thickness (mm)**	**MT**	0.37 [0.29,0.44]	0.35 [0.27,0.42]	0.7422†
	**LT**	0.36 [0.29,0.44]	0.36 [0.29,0.46]	0.8203†
	**MF**	0.36 [0.30,0.41]	0.36 [0.33,0.40]	0.6753†
	**LF**	0.36 [0.28,0.45]	0.39 [0.31,0.47]	0.4911ˣ
**Cortical Porosity (%)**	**MT**	1.81 [-0.63,4.26]	2.656 [-0.12,5.42]	0.8438†
	**LT**	1.86 [-0.78,4.51]	1.04 [-0.42,2.50]	0.1562†
	**MF**	3.83 [-2.99,10.64]	1.45 [-0.67,3.57]	0.1953†
	**LF**	0.33 [0.29,0.36]	0.32 [0.30,0.34]	0.7439ˣ
