# Supplementary material for: Early removal of the infrapatellar fat pad/synovium complex beneficially alters the pathogenesis of moderate stage idiopathic knee osteoarthritis in male Dunkin Hartley guinea pigs
Source: Arthritis Res Ther. 2022 Dec 28;24:282. doi: 10.1186/s13075-022-02971-y (PMC9795160; doi:10.1186/s13075-022-02971-y)
Supplement: Supplementary file 1 — Additional file 1. Supplementary material. [file 13075_2022_2971_MOESM1_ESM.zip › Supplemental Table 8. Meniscus and Cartilage Indentation analysis_ESM.pdf]

Supplemental Table 8. Meniscus and Cartilage Indentation Analysis. Mean values (with 95% confidence interval) for Biomechanical measurements for IFP/SC vs FCT limbs. Normally distributed data with similar variance were compared using parametric ratio t tests†. Data with non-Gaussian distribution were compared using non-parametric Wilcoxon matched – pairs signed rank test ×.

		IFP/SC (sham)	FCT (IFP removal)	P-value
**Meniscus Indentation**	**MA** I. Modulus (MPa)	36.01 [16.39,55.64]	31.44 [17.82,45.06]	0.8783†
	**MP** I. Modulus (MPa)	16.04 [8.06,24.01]	17.53 [8.73,26.33]	0.7268†
	**MA** E. Modulus (MPa)	5.32 [1.48,9.16]	6.57 [0.39, 12.75]	0.9375×
	**MP** E. Modulus (MPa)	1.16 [0.76, 1.57]	0.55 [0.07,1.02]	***0.0245†***
	**LA** I. Modulus (MPa)	32.32 [8.89,55.76]	23.36 [3.49,43.23]	0.5410†
	**LP** I. Modulus (MPa)	18.85 [6.33,31.36]	17.16 [9.26, 25.05]	0.9506†
	**LA** E. Modulus (MPa)	7.54 [-1.99,17.06]	3.28 [-0.20, 6.77]	0.4865†
	**LP** E. Modulus (MPa)	1.38 [0.58,2.18]	1.31 [0.53,2.09]	0.8744†
**Cartilage Indentation**	**MT** I. Modulus (MPa)	19.64 [13.06,26.23]	21.31 [-1.09,43.71]	0.6875×
	**MF** I. Modulus (MPa)	62.55 [31.52,93.59]	44.60 [25.39,63.80]	0.2065†
	**MT** E. Modulus (MPa)	6.05 [1.71,10.39]	2.20 [0.34,4.05]	***0.0413†***
	**MF** E. Modulus (MPa)	18.84 [6.11,31.57]	6.83 [-0.13,13.78]	***0.0402†***
	**LT** I. Modulus (MPa)	23.85 [16.39,31.32]	45.74 [2.24,89.24]	0.5781×
	**LF** I. Modulus (MPa)	66.67 [29.01,104.30]	61.31 [27.48,95.13]	0.8290†
	**LT** E. Modulus (MPa)	3.37 [2.59,4.16]	6.74 [0.81,12.68]	0.3617†
	**LF** E. Modulus (MPa)	25.36 [8.79,41.94]	16.98 [-2.03,35.98]	0.2373†

Instaneous Modulus (I. Modulus); Equilibrium Modulus (E. Modulus); MA, medial anterior; MP, medial posterior; LA, lateral anterior; LP, lateral posterior; MT, medial tibia; LT, lateral tibia; MF, medial femur; LF, lateral femur.
