# Supplementary material for: Early removal of the infrapatellar fat pad/synovium complex beneficially alters the pathogenesis of moderate stage idiopathic knee osteoarthritis in male Dunkin Hartley guinea pigs
Source: Arthritis Res Ther. 2022 Dec 28;24:282. doi: 10.1186/s13075-022-02971-y (PMC9795160; doi:10.1186/s13075-022-02971-y)
Supplement: Supplementary file 1 — Additional file 1. Supplementary material. [file 13075_2022_2971_MOESM1_ESM.zip › Supplemental Table 9 & 10. Cartilage AAS_ESM.pdf]

Supplemental Table 9. Atomic Absorption Spectroscopy (AAS) trace element concentrations of Medial Cartilage. Mean values (with 95% confidence interval) for medial cartilage (femur & tibia) for IFP/SC vs FCT limbs. Normally distributed data with similar variance were compared using parametric ratio t-tests[†]. Data with non-Gaussian distribution were compared using non-parametric Wilcoxon matched – pairs signed rank test [×].

	Concentration (ppm)		
**Trace Element**	**Left Hind (IFP/SC)**	**Right Hind (FCT)**	**P-value**
**Calcium** (Ca)	106738<br>[75623, 137852]	100300<br>[75750, 124850]	0.5342[†]
**Magnesium** (Mg)	2018<br>[1626, 2409]	1641<br>[1387, 1895]	***0.0168**[†]
**Zinc** (Zn)	141.8<br>[93.5, 190.2]	107.5<br>[78.72, 136.2]	0.1094[×]
**Iron** (Fe)	77.63<br>[42.8, 112.4]	72.89<br>[52.17, 93.6]	0.9269[×]
**Phosphorous** (P)	30732<br>[15923, 44820]	23847<br>[14206, 33487]	***0.0547**[×]

Supplemental Table 10. Atomic Absorption Spectroscopy (AAS) trace element concentrations of Lateral Cartilage. Mean values (with 95% confidence interval) for lateral cartilage (femur and tibia) for IFP/SC vs FCT limbs. Normally distributed data with similar variance were compared using parametric ratio t-tests[†]. Data with non-Gaussian distribution were compared using non-parametric Wilcoxon matched – pairs signed rank test [×].

	Concentration (ppm)		
**Trace Element**	**Left Hind (IFP/SC)**	**Right Hind (FCT)**	**P-value**
**Calcium** (Ca)	112038<br>[78700, 145000]	100900<br>[49900, 151000]	0.2139[†]
**Magnesium** (Mg)	2345<br>[1910, 3020]	2180<br>[1230,3130]	0.2323[†]
**Zinc** (Zn)	246<br>[184, 391]	162.6<br>[129, 223]	****0.0078**[×]
**Iron** (Fe)	71.45<br>[35.2, 120]	88.75<br>[47.5, 145]	0.4039[×]
**Phosphorous** (P)	82575<br>[27600, 157000]	86213<br>[45500, 171000]	0.8438[×]
