# Supplementary material for: Early removal of the infrapatellar fat pad/synovium complex beneficially alters the pathogenesis of moderate stage idiopathic knee osteoarthritis in male Dunkin Hartley guinea pigs
Source: Arthritis Res Ther. 2022 Dec 28;24:282. doi: 10.1186/s13075-022-02971-y (PMC9795160; doi:10.1186/s13075-022-02971-y)
Supplement: Supplementary file 1 — Additional file 1. Supplementary material. [file 13075_2022_2971_MOESM1_ESM.zip › Supplemental Table S7. Femur Trabecular_subchondral AAS.pdf]

Supplemental Table 7. Cortical/Subchondral Trabecular bone AAS. Means values (with 95% confidence interval) for trace element concentrations from tissue collected from medial/lateral distal femoral condyles from IFP/SC and FCT hind limbs. Normally distributed data with similar variance were compared using parametric ratio t tests[†]. Data with non-Gaussian distribution were compared using non-parametric Wilcoxon matched – pairs signed rank test[×].

Trace Element	Compartment	IFP/SC	FCT	P-value
**Calcium** (Ca)	**MF**	255,500<br>[245,639;265,361]	252,667<br>[243,599;261,735]	0.0777[†]
	**LF**	239,875<br>[231,938;247,812]	233,750<br>[224,939;242,561]	0.2344[×]
**Magnesium** (Mg)	**MF**	4,674<br>[4,369;4,978]	4,494<br>[4,201;4,787]	**0.0059[†]
	**LF**	4,400<br>[4,047;4,753]	4,140<br>[3,780;4,500]	0.0787[†]
**Zinc** (Zn)	**MF**	252.3<br>[222.5;282.0]	258.4<br>[222.3;294.4]	0.8030[†]
	**LF**	248.1<br>[206.4;289.9]	238.4<br>[199.7;277.0]	0.6964[†]
**Iron** (Fe)	**MF**	59.73<br>[43.12, 76.33]	65.01<br>[52.43;77.60]	0.1875[×]
	**LF**	81.19<br>[43.60;118.8]	93.44<br>[56.92; 130.0]	0.5469[×]
**Phosphorous** (P)	**MF**	115,238<br>[105,704;124,771]	124,500<br>[120,656;128,344]	*0.0469[×]
	**LF**	124,350<br>[87,704;160,996]	112,800<br>[95,817;129,783]	0.8203[×]

Medial Femur (MF); Lateral Femur (LF)
