# Supplementary figures and images for: Early removal of the infrapatellar fat pad/synovium complex beneficially alters the pathogenesis of moderate stage idiopathic knee osteoarthritis in male Dunkin Hartley guinea pigs
Source: Arthritis Res Ther. 2022 Dec 28;24:282. doi: 10.1186/s13075-022-02971-y (PMC9795160; doi:10.1186/s13075-022-02971-y)

**A****Body Weight**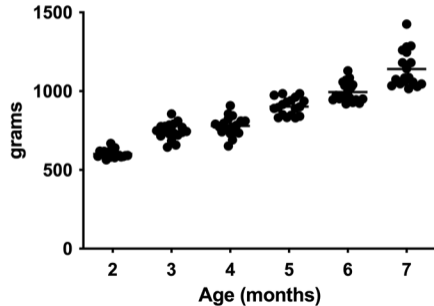**B****Body Weight**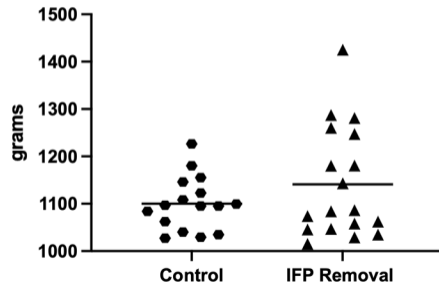**C****Femur Length**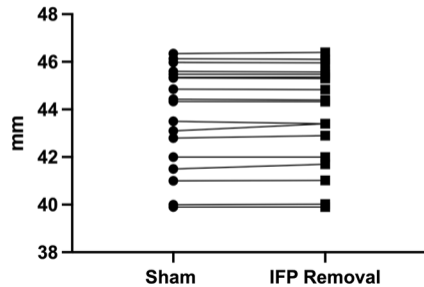

Supplement: Supplementary file 1 — Additional file 1. Supplementary material. [file 13075_2022_2971_MOESM1_ESM.zip › Supplemental Figure 1. Guinea pig description.pdf]

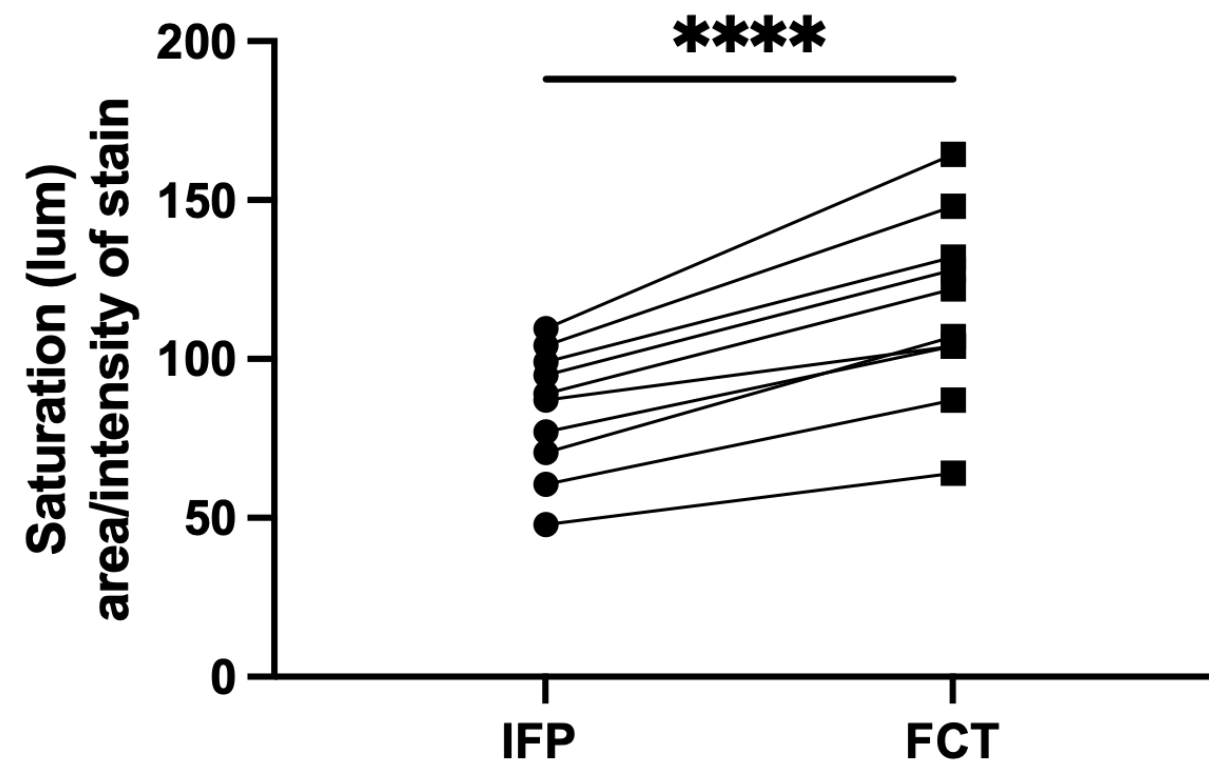

Supplement: Supplementary file 1 — Additional file 1. Supplementary material. [file 13075_2022_2971_MOESM1_ESM.zip › Supplemental Figure 2. Masson's Trichrome quanitation.pdf]
